# Effect of nonlinear thermal radiation and Cattaneo-Christov heat and mass fluxes of Williamson hybrid nanofluid over a stretching porous sheet

**DOI:** 10.12688/f1000research.160734.3

**Published:** 2025-12-03

**Authors:** Asfaw Tsegaye, Eshetu Haile, Gurju Awgichew, Hunegnaw Dessie

**Affiliations:** 1 Bahir Dar University Department of Mathematics, Bahir Dar, Amhara, Ethiopia

**Keywords:** Williamson fluid, Hybrid nanofluid, Cattaneo-Christov heat and mass flux, non-linear thermal radiation, stretching sheet

## Abstract

**Background:**

Hybrid nanofluids, consisting of two distinct nanoparticles dispersed in a base fluid, are widely used in industries requiring enhanced heat and mass transfer, such as cooling systems and heat exchangers. These fluids improve thermal conductivity and fluid dynamics, leading to better heat management and energy efficiency. This study investigates the combined effects of non-linear thermal radiation, Cattaneo-Christov heat and mass fluxes, and other factors on the three-dimensional flow, heat, and mass transfer of a Williamson hybrid nanofluid. The flow occurs over a stretching porous sheet subjected to an external magnetic field, Joule heating, chemical reactions, and heat generation.

**Methods:**

Copper (Cu) and aluminum oxide (Al₂O₃) nanoparticles are suspended in ethylene glycol (C₂C₆O₂) to form the hybrid nanofluid. The governing partial differential equations are transformed into ordinary differential equations using similarity transformations and solved numerically with MATLAB’s bvp4c solver. The study examines various parameters, including stretching ratio, nanoparticle volume fraction, and relaxation times for concentration and thermal effects. Results are validated against existing literature.

**Results:**

The findings reveal that a higher stretching ratio reduces velocity, temperature, concentration profiles, and local Nusselt and Sherwood numbers, while also lowering skin friction and secondary velocity. Increasing nanoparticle volume fraction decreases velocity and temperature profiles but enhances skin friction, local Nusselt, and Sherwood numbers. Concentration profiles decline with higher concentration relaxation time, while temperature increases with longer thermal relaxation time.

**Conclusions:**

In conclusion, Cu−Al₂O₃/C₂C₆O₂ hybrid nanofluids demonstrate superior heat and mass transfer capabilities compared to mono-nanofluids. The performance is significantly influenced by parameters such as nanoparticle volume fraction, relaxation times, and the stretching ratio, providing valuable insights for heat and mass transfer applications.

**Nomenclature and notations**
*A*_1_
: First Rivlin-Erickson tensor [Pa]
*B*_0_
: Uniform magnetic field [T]
*Q*
: Constant volumetric heat source/sink [W/m ^3^]
*B*
: External magnetic field [T]
*C _p_*
: Specific heat [J/kg·K]
*u, v, w*
: Velocity components [m/s]
*T _w_*
: Wall temperature [K]
*T*_∞_
: Temperature of the ambient fluid [K]
*C _w_*
: Wall Concentration [mol/m ^3^]
*C*_∞_
: Concentration of the ambient fluid
*d*
: Stretching ratio parameter
*C _f_*
: Skin friction
*R*
: Non-linear thermal radiation parameter
*K*
: Porosity parameter
*H*
: Chemical reaction [s ^−1^]
*Sc*
: Schmidt number
*x, y, z*
: Space coordinates [m]
*Ec*
: Eckert number
*D _B_*
: Coefficient of Brownian diffusion [m ^2^/s]
*D _T_*
: Coefficient of thermophoresis diffusion [m ^2^/s]
*I*
: Identity vector
*M*
: Magnetic field parameter

$\vec{q}$

: Heat flux

$\vec{j}$

: Mass flux
*Nt*
: Thermophoresis parameter
*T*
: Fluid Temperature
*q _r_*
: Nonlinear radiative heat flux
*C*
: Fluid Concentration

$\vec{V}$

: Velocity vector [m/s]

$\vec{S}$

: extra stress tensor [Pa]
*P*
: Pressure [Pa]
*Nb*
: Brownian motion parameter
*Nu _x_*
: Nusselt number
*Pr*
: Prandtl number
*Sh _x_*
: Local Sherwood number
*We*
: Williamson fluid parameter
*κ _f_*
: Thermal conductivity of base fluid
*u _w_*
: Velocity of Stretching Sheet along x-axis [m/s]
*v _w_*
: Velocity of Stretching Sheet along y-axis [m/s]
*μ*_∞_
: Limiting viscosity at infinite
*κ* ^∗^
: Rosseland mean absorption coefficient [1/m]

$\theta_{w}$

: Ratio temperature
Γ: Time constant
( *ρC _p_*) *_f_*
: Base fluid heat capacity

θ

: Dimensionless temperature
Φ: Dimensionless concentration function
*ν _hnf_*
: Kinematic viscosity of hybrid nanofluid [m ^2^/s]

$f^{\prime }$

: Primary Dimensionless velocity
( *ρC _p_*) *_p_*
: Heat capacity of a nanoparticle [J/m ^3^·K]
*g* ^r^
: Secondary Dimensionless velocity
( *ρC _p_*) *_hnf_*
: Hybrid nanofluid heat capacitance [J/m ^3^·K]
*σ _hnf_*
: Hybrid nanofluid electrical conductivity [S/m]
*ρ _hnf_*
: Density of hybrid nanofluid [kg/m ^3^]
*κ _hnf_*
: Thermal conductivity of hybrid nanofluid [W/m·K]
∞: Ambient condition
*κ*
: Thermal conductivity [W/m·K]
*η*
: Similarity variable
*α _hnf_*
: Thermal diffusivity of hybrid nanofluid
*μ _f_*
: Base fluid of dynamic viscosity [Pa·s]
*σ* ^∗^
: Stefan Boltzmann constant [W/m ^2^·K ^4^]
*μ _hnf_*
: Hybrid nanofluid dynamic viscosity [Pa·s]
*ρ _f_*
: Density of base fluid

$\phi_{1}$

: Nanoparticle volume fraction of Copper
*μ*_0_
: Limiting viscosity at zero shear rate [Pa·s]
*β*_1_
: Thermal relaxation parameter [s]
*β*_2_
: Concentration relaxation parameter [s]
*λ _e_*
: Energy relaxation factor [s]
*λ _c_*
: Concentration relaxation factor [s]
*τ*
: Ratio of heat capacity
*Q*_0_
: Heat generation ( *Q*_0_ *>* 0) and absorption [W/m ^3^]
( *σ*_*f*_): Electrical conductivity of base fluid
*Re _x_*
: Local Reynolds number

$\phi_{2}$

: Nanoparticle volume fraction of Aluminum Oxide

## 1. Introduction

Nowadays, researchers have created novel fluids to suit the requirement for improved heat transmission and thermal conductivity. The heat transfer properties of conventional fluids like water, ethylene glycol, glycerin, and ethanol are limited in applications such as power generation, chemical processes, and heating and cooling systems. Scientists are investigating better heat transmission materials to meet the rising energy demands and address concerns about shortages of resources and environmental impacts. A single kind of nanoparticle can be added to the aforementioned fluids to make up for their deficiency. The term nanofluid refers to this process, which was initially studied by Choi and Eastman. ^1^ Researchers have considered many combinations of nanoparticles, including semiconductors ( *SiO*_2_ *, TiO*_2_), metallic oxides ( *Al*_2_ *O*_3_ *, CuO*), and metal nanoparticles ( *Al*, *Cu*, and *Fe*). Heat transfer through nanofluids has been investigated from various perspectives. Mahian et al. ^2^ presented the uses of nanofluid in a variety of contexts, such as renewable energy systems. They also covered the advantages of energy systems from an environmental perspective when employing nanofluid. Mansoury et al. ^3^ investigated the flow of *Al*_2_ *O*_3_ */H*_2_ *O* nanofluids through parallel heat exchangers. Unfortunately, to obtain the required thermal performance, a single nanoparticle suspension is insufficient. To attain the required thermal properties hybrid nanofluid is employed. Several experimental and theoretical models have been published and studied in order to use hybrid nanofluids for more efficient industrial and technological processes. Mahanthesh et al. ^4^ reviewed the flow behavior of hybrid nanofluids, focusing on the effects of Brownian motion and thermophoresis. Sensitivity analysis revealed that the Brownian motion parameter has the most significant impact on the heat transfer rate. Bilal et al. ^5^ investigated the Darcy-Forchheimer mixed convection flow of hybrid nanofluids through an inclined, extending cylinder using the Homotopy analysis method. Their findings indicated that CNT−Fe _3_O _4_/H _2_O hybrid nanofluids enhance the thermal efficiency of the base fluid more effectively than conventional fluids. Jat et al. ^6^ investigated the impact of various nanoparticle shapes on the radiating hybrid nanofluid flow, particularly over a nonlinear stretchable porous sheet. Faisal et al. ^7^ numerically examined Casson hybrid nanofluid flow over a bidirectionally stretching surface in a stagnation region, incorporating entropy generation analysis. The governing equations were transformed using similarity variables and solved through an appropriate numerical scheme to assess the effects of key physical parameters on flow, heat transfer, and entropy production. The investigation provides further information on the hybrid nanofluid flow as provided in these references. ^8, 9^

Scientists and engineers have been motivated by the non-Newtonian motion of fluids because these materials have multiple applications in science and technology processes. Some examples of non-Newtonian fluid are Paints, mud, Soap, glue, apple sauce, printing ink, shampoos, sugar solutions, tomato paste, etc. Furthermore, non-Newtonian fluids are used in several kinds of fields of study, such as chemical engineering field, biology, and geophysical sciences. The Williamson model is the fluid model that is being studied. In the case of Williamson fluid rheology, the constitutive equation for stress-strain is non-linear. Therefore, viscosity that depends on the shear rate forms the basis of the Williamson fluid model. This model captures both shear-thinning and shear-thickening behavior. Williamson ^10^ introduced the Williamson fluid model, which defines the relationship between stress and strain in pseudoplastic materials. Kebede et al. ^11^ studied the heat and mass transport properties of Williamson nanofluid flow. Kumar et al. ^12^ explored the impact of Brownian motion and thermophoresis on heat and mass transfer in pseudoplastic materials. Reddy et al. ^13^ investigated the impact of Cattaneo-Christov heat flux and suction/injection on the entropy generation in the system. Chakradhar et al. ^14^ examined the influence of MHD effects on the peristaltic motion of Williamson fluid within a porous channel. Faisal et al. ^15^ explored the bidirectional flow and heat transfer characteristics of a hyperbolic tangent hybrid nanofluid under Nield’s conditions. Using similarity transformations and a numerical solution approach, the study examined how key physical parameters influence the nanofluids velocity, temperature distribution, and heat transfer behavior.

Heat and mass transport are natural phenomena caused by concentration and temperature differences within or between materials. Many industrial processes, including wire drawing, artificial fiber production, paper manufacturing, chemical waste migration, and distillation, are influenced by this phenomenon. Significant efforts have been made in the past to study heat and mass transport mechanisms using Fourier’s law of heat transfer ^16^ and Fick’s law of diffusion. ^17^ Previously, “Fourier’s law of heat conduction” was commonly used to explain heat transfer. However, this law does not fully capture the fundamental nature of heat transfer. To address this issue, Cattaneo ^18^ introduced thermal relaxation into Fourier’s theory, making heat transfer resemble thermal wave propagation at normal speed. Christov ^19^ improved the Cattaneo model’s thermal relaxation time by using upper-convected Oldroyd derivatives for frame-invariant formation. Sui et al. ^20^ extended the Cattaneo-Christov model to mass diffusion problems, applying it to the Maxwell nanofluid mass diffusion across a moving surface. Vinodkumar et al. ^21^ explored the behavior of MHD convective flow of non-Newtonian nanofluids over a chemically reacting porous sheet. Vinodkumar et al. ^22^ examined the effects of higher-order chemical reactions and radiation on the MHD flow of a Maxwell nanofluid. Recent developments on this concept are gathered. ^23– 28^

Magnetohydrodynamics is the study of the interaction between a magnetic field and electrically conducting fluids, such as saltwater solutions, liquid metals, and plasmas. Magnetohydrodynamic flow studies have significant applications in chemistry, physics, and engineering. Additionally, MHD flow has applications in industrial equipment, pumps, electric transformers, and hydro-magnetic generators, among others. Zhao et al. ^29^ studied the thermally induced electro-kinetic flow of Al _2_O _3_-water nanofluid through a permeable micro-tube to examine its heat transfer properties, considering the effect of an applied magnetic field. Chamkha et al. ^30^ used the finite-difference method to analyze mixed convection in a square cavity filled with Cu-water nanofluid. MS et al. ^31^ analyzed the entropy optimization in the MHD flow of non-Newtonian nanofluids with chemical reactions and thermal energy effects. Faisal et al. ^32^ analyzed heat and mass transfer in a 3D Maxwell-slip flow of a water-based ternary hybrid nanofluid subject to Smoluchowski-Nield constraints. Their model incorporates Brownian motion, thermophoresis, thermal radiation, and wall slip, with the governing equations reduced via similarity transformations and solved using an iterative numerical scheme with convergence and error analysis. Vinodkumar et al. ^33^ investigated the activation and behavior of energy in the magnetohydrodynamic (MHD) flow of Casson nanofluids through a porous medium. Recent studies on MHD can be found in references. ^34– 37^

Flow over a stretching sheet is a classic fluid mechanics problem with various practical applications in industries such as glass fiber production, glass blowing, wire drawing, and copper wire tinning. The concept of boundary layer flow for solid surface motion at a constant speed was first introduced by Sakiadis. ^38– 40^ Several researchers have recently investigated the flow of boundary layers over stretchable surfaces. ^41, 42^

In fluid dynamics, a porous medium refers to a solid material that contains a network of interconnected voids or pores, allowing fluids (such as gases or liquids) to flow through it. The structure of the porous medium, including the size, shape, and distribution of its pores, significantly influences the flow behavior and other transport phenomena (e.g., heat and mass transfer) within the medium. Shaw et al. ^43^ investigated the effects of entropy production in a Casson fluid containing *MWCNT/Fe*_3_ *O*_4_ through a stretched disc and the Darcy-Forchheimer porous medium concept using a numerical approach. There are further investigations on the porous media. ^44, 45^

Nonlinear thermal radiation refers to the heat transfer process where the radiation heat flux does not follow a simple linear relationship with temperature. Unlike linear radiation, where the heat flux is proportional to the fourth power of temperature (as in the Stefan-Boltzmann law), nonlinear thermal radiation accounts for more complex interactions between the radiating bodies and the surrounding medium. This phenomenon arises in high-temperature environments, where the radiation intensity varies non-linearly due to factors such as temperature gradients, optical properties of materials, or the nature of the medium. Alamirew et al. ^46^ studied the impact of nonlinear thermal radiation, ion slip, and Hall on MHD Williamson nanofluid flow over a stretched sheet. Building on this, other researchers ^47, 48^ have explored the effects of thermal radiation in various geometries and methods.

Several studies have investigated Williamson nanofluid flow over a stretching sheet using various geometries and methods. Different approaches have been employed in the literature to study the heat and mass fluxes in Williamson nanofluid flow. However, to the best of the authors’ knowledge, no studies have yet considered the combined effects of non-linear thermal radiation, Joule heating, non-Fourier heat flux, non-Fick mass flux, chemical reactions, heat generation/absorption, Brownian motion, and thermophoresis. The present study aims to provide a comprehensive analysis of mass and heat transfer in 3D MHD Williamson hybrid nanofluid flow, which consists of Cu and Al _2_O _3_ nanoparticles in ethylene glycol, over a linearly stretching porous sheet. By applying similarity transformations, the governing nonlinear partial differential equations are converted into a system of ordinary differential equations. The reduced mathematical model is then solved using the bvp4c function in MATLAB. The study uses tables and figures to analyze the effects of various parameters, such as velocity, temperature, concentration, skin friction, the Nusselt number, and the Sherwood number. The findings of this study have important implications for engineering and industrial applications, including aerospace engineering, nuclear power plants, electronic cooling, biomedical systems, and the polymer and textile industries.

## 2. Problem formulation

**Figure 1.**
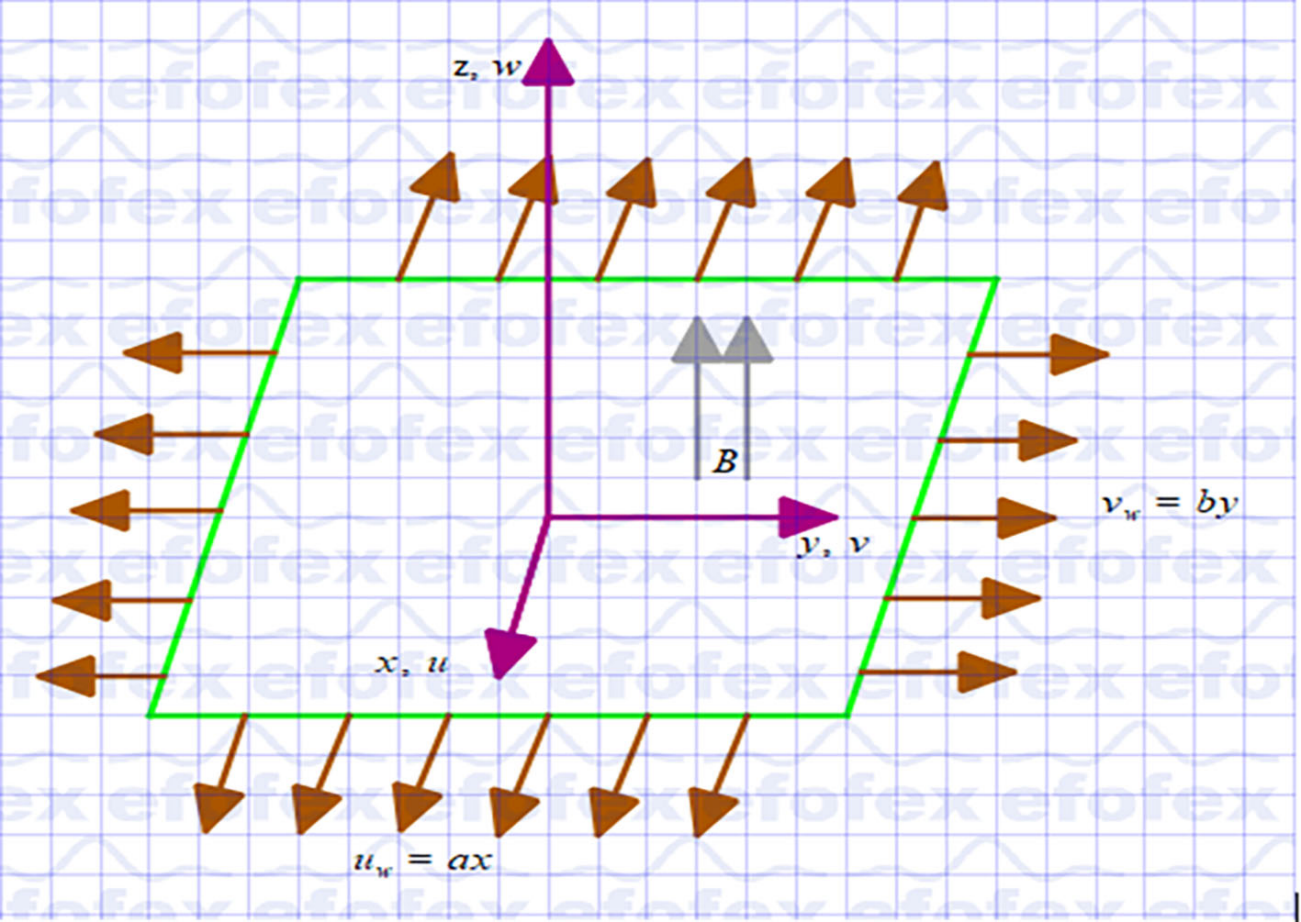
Coordinate system.

Assume a steady three-dimensional hybrid nanofluid ( $\mathrm{Cu}-{\mathrm{Al}}_{2}O_{3}$ /Ethylene glycol) flow-past a stretching porous sheet in the presence of an applied magnetic field. The schematic representation in Cartesian coordinates is displayed in Figure 1. The flow is incompressible and laminar. Cattaneo-Christov Heat and Mass Flux Model, Non-Linear Thermal Radiation, Joule Heating, Viscous Dissipation, Porous Medium and Magnetic Field Effects, heat generation, and chemical reaction are applied. The velocities of the stretching sheets are $v_{w}=\mathrm{by}$ in y and $u_{w}=\mathrm{ax}$ in *x*, and a constant external magnetic field B is applied in z . The temperature and concentration of the surface are kept constant at $T_{w}$ and $C_{w}$ respectively, which is higher than its ambient temperature, $T_{\infty }$ and ambient concentration, $C_{\infty }$ . Furthermore, the double diffusion Cattaneo-Christov theory generalization of the well-known Fourier and Fick laws was used to study heat and mass transmission. ^49, 50^

$$\vec{q}+\kappa \nabla T+\lambda_{e}\left[{\vec{q}}_{t}+\vec{V}\cdot \nabla \vec{q}-\vec{q}\cdot \nabla \vec{V}+\left(\nabla \cdot \vec{V}\right)\vec{q}\right]=0.$$ (1)

$$\vec{J}+D_{B}\nabla C+\lambda_{c}\left[{\vec{J}}_{t}+\vec{V}\cdot \nabla \vec{J}-\vec{J}\cdot \nabla \vec{V}+\left(\nabla \cdot \vec{V}\right)\vec{J}\right]=0.$$ (2)

In equation (1) and (2), $\vec{q}$ and $\vec{J},D_{B},T,C,\kappa ,\vec{V},\lambda_{e}$ and $\lambda_{c}$ represents the heat and mass fluxes, the Brownian motion, the temperature, the concentration, the thermal conductivity, the velocity field, the energy and concentration relaxation factors, respectively. By letting $\lambda_{e}=0=\lambda_{c}$ , the famous Fourier’s and Fick’s laws can be obtained. From equations (1) and (2), which generalize the classic Fourier and Fick’s laws, the steady-state conditions of the nanofluid flow are expressed as follows:

$$\vec{q}+\kappa \nabla T+\lambda_{e}\left[\vec{V}\cdot \nabla \vec{q}-\vec{q}\cdot \nabla \vec{V}+\left(\nabla \cdot \vec{V}\right)\vec{q}\right]=0.$$ (3)

$$\vec{J}+D_{B}\nabla C+\lambda_{c}\left[\vec{V}\cdot \nabla \vec{J}-\vec{J}\cdot \nabla \vec{V}+\left(\nabla \cdot \vec{V}\right)\vec{J}\right]=0.$$ (4)

The Williamson fluid model is given Refs. 51, 52 by

$$\tau =-\vec{P}I+\vec{S}$$ (5)

$$\vec{S}=\left[\mu_{\infty }+\frac{\mu_{0}-\mu_{\infty }}{1-\Gamma \dot{\gamma }}\right]A_{1}.$$ (6)

The shear rate ( $\dot{\gamma }$ ) and is defined as

$$\dot{\gamma }=\sqrt{0.5\text{trace}{\left(A_{1}\right)}^{2}}.$$ (7)

This is taken into consideration for pseudo-plastic fluids $\mu_{\infty }=0$ and $\Gamma \dot{\gamma }<1$ . Equation (6) can be written as

$$\vec{S}=\left[\mu_{0}/\left(1-\Gamma \dot{\gamma }\right)\right]A_{1}.$$ (8)

Using the binomial expansion to Equation (8), given as

$$\vec{S}\approx \mu_{0}\left[1+\Gamma \dot{\gamma }\right]A_{1}.$$ (9)

Considering the above assumptions, and after applying the boundary layer approximations, the governing equations are expressed as follows ^49, 53, 54^

$$\frac{\partial u}{\partial x}+\frac{\partial v}{\partial y}+\frac{\partial w}{\partial z}=0,$$ (10)

$$u\frac{\partial u}{\partial x}+v\frac{\partial u}{\partial y}+w\frac{\partial u}{\partial z}=\frac{\mu_{\mathrm{hnf}}}{\rho_{\mathrm{hnf}}}\frac{\partial^{2}u}{\partial z^{2}}+\sqrt{2}\Gamma \frac{\mu_{\mathrm{hnf}}}{\rho_{\mathrm{hnf}}}\frac{\partial u}{\partial z}\frac{\partial^{2}u}{\partial z^{2}}-\frac{\sigma_{\mathrm{hnf}}B^{2}}{\rho_{\mathrm{hnf}}}u-\frac{\mu_{\mathrm{hnf}}}{\rho_{\mathrm{hnf}}K_{p}}u,$$ (11)

$$u\frac{\partial v}{\partial x}+v\frac{\partial v}{\partial y}+w\frac{\partial v}{\partial z}=\frac{\mu_{\mathrm{hnf}}}{\rho_{\mathrm{hnf}}}\frac{\partial^{2}v}{\partial z^{2}}+\sqrt{2}\Gamma \frac{\mu_{\mathrm{hnf}}}{\rho_{\mathrm{hnf}}}\frac{\partial v}{\partial z}\frac{\partial^{2}v}{\partial z^{2}}-\frac{\sigma_{\mathrm{hnf}}B^{2}}{\rho_{\mathrm{hnf}}}v-\frac{\mu_{\mathrm{hnf}}}{\rho_{\mathrm{hnf}}K_{p}}v,$$ (12)

$$u\frac{\partial T}{\partial x}+v\frac{\partial T}{\partial y}+w\frac{\partial T}{\partial z}=\alpha_{\mathrm{hnf}}\frac{\partial^{2}T}{\partial z^{2}}-\frac{1}{{\left(\mathrm{\rho Cp}\right)}_{\mathrm{hnf}}}\frac{\partial q_{r}}{\partial z}-\lambda_{e}[u^{2}\frac{\partial^{2}T}{\partial x^{2}}+v^{2}\frac{\partial^{2}T}{\partial y^{2}}+w^{2}\frac{\partial^{2}T}{\partial z^{2}}+2\left(\mathrm{uv}\frac{\partial^{2}T}{\partial x\partial y}+\mathrm{vw}\frac{\partial^{2}T}{\partial y\partial z}+\mathrm{uw}\frac{\partial^{2}T}{\partial x\partial z}\right)+\left(u\frac{\partial u}{\partial x}+v\frac{\partial u}{\partial y}+w\frac{\partial u}{\partial z}\right)\frac{\partial T}{\partial x}+\left(u\frac{\partial v}{\partial x}+v\frac{\partial v}{\partial y}+w\frac{\partial v}{\partial z}\right)\frac{\partial T}{\partial y}+\left(u\frac{\partial w}{\partial x}+v\frac{\partial w}{\partial y}+w\frac{\partial w}{\partial z}\right)\frac{\partial T}{\partial z}]+\frac{Q_{0}\left(T-T_{\infty }\right)}{{\left(\mathrm{\rho Cp}\right)}_{\mathrm{hnf}}}+\frac{\sigma_{\mathrm{hnf}}B^{2}\left(u^{2}+v^{2}\right)}{{\left(\mathrm{\rho Cp}\right)}_{\mathrm{hnf}}}+\tau \left[D_{B}\frac{\partial C}{\partial z}\frac{\partial T}{\partial z}+\frac{D_{T}}{T_{\infty }}{\left(\frac{\partial T}{\partial z}\right)}^{2}\right],$$ (13)

$$u\frac{\partial C}{\partial x}+v\frac{\partial C}{\partial y}+w\frac{\partial C}{\partial z}+\kappa_{c}\lambda_{c}=D_{B}\frac{\partial^{2}C}{\partial z^{2}}+\frac{D_{T}}{T_{\infty }}\left(\frac{\partial^{2}T}{\partial z^{2}}\right)-K_{r}\left(C-C_{\infty }\right)-\lambda_{c}[u^{2}\frac{\partial^{2}C}{\partial x^{2}}+v^{2}\frac{\partial^{2}C}{\partial y^{2}}+w^{2}\frac{\partial^{2}C}{\partial z^{2}}+2\left(\mathrm{uv}\frac{\partial^{2}C}{\partial x\partial y}+\mathrm{vw}\frac{\partial^{2}C}{\partial y\partial z}+\mathrm{uw}\frac{\partial^{2}C}{\partial x\partial z}\right)+\;\left(u\frac{\partial u}{\partial x}+v\frac{\partial u}{\partial y}+w\frac{\partial u}{\partial z}\right)\frac{\partial C}{\partial x}+\left(u\frac{\partial v}{\partial x}+v\frac{\partial v}{\partial y}+w\frac{\partial v}{\partial z}\right)\frac{\partial C}{\partial y}+\left(u\frac{\partial w}{\partial x}+v\frac{\partial w}{\partial y}+w\frac{\partial w}{\partial z}\right)\frac{\partial C}{\partial z}].$$ (14)

The boundary conditions for the problem are given by ^53, 55^

$$\left\{ \begin{array}{l} w=0,v=v_{w}=\mathrm{by},u=u_{w}=\mathrm{ax},T=T_{w},C=C_{w},\mathrm{at}\;z\to 0\\ u=v=0,T=T_{\infty },C=C_{\infty },\mathrm{as}\;z\to \infty . \end{array}\right.$$ (15)

The hybrid nanofluids thermophysical characteristics are described as ^52, 53^:

$$\begin{array}{c} \mu_{\mathrm{hnf}}=\frac{\mu_{f}}{{\left(1-\phi_{1}\right)}^{2.5}{\left(1-\phi_{2}\right)}^{2.5}},\alpha_{\mathrm{hnf}}=\frac{\kappa_{\mathrm{hnf}}}{{\left(\mathrm{\rho Cp}\right)}_{\mathrm{hnf}}},\\ \rho_{\mathrm{hnf}}=\left(1-\phi_{2}\right)\left[\left(1-\phi_{1}\right)\rho_{f}+\phi_{1}\rho_{n1}\right]+\phi_{2}\rho_{n2},\\ {\left(\mathrm{\rho Cp}\right)}_{\mathrm{hnf}}=\left(1-\phi_{2}\right)\left[\left(1-\phi_{1}\right){\left(\mathrm{\rho Cp}\right)}_{f}+\phi_{1}{\left(\mathrm{\rho Cp}\right)}_{n1}\right]+\phi_{2}{\left(\mathrm{\rho Cp}\right)}_{n2},\\ \frac{\kappa_{\mathrm{hnf}}}{\kappa_{\mathrm{nf}}}=\frac{\kappa_{n2}+2\kappa_{\mathrm{nf}}-2\phi_{2}\left(\kappa_{\mathrm{nf}}-\kappa_{n2}\right)}{\kappa_{n2}+2\kappa_{\mathrm{nf}}+\phi_{2}\left(\kappa_{\mathrm{nf}}-\kappa_{n2}\right)},\frac{\kappa_{\mathrm{nf}}}{\kappa_{f}}=\frac{\kappa_{n1}+2\kappa_{f}-2\phi_{1}\left(\kappa_{f}-\kappa_{n1}\right)}{\kappa_{n}+2\kappa_{f}+\phi \left(\kappa_{f}-\kappa_{n1}\right)},\\ \frac{\sigma_{\mathrm{hnf}}}{\sigma_{\mathrm{nf}}}=\frac{\sigma_{n2}+2\sigma_{\mathrm{nf}}-2\phi_{2}\left(\sigma_{\mathrm{nf}}-\sigma_{n2}\right)}{\sigma_{n2}+2\sigma_{\mathrm{nf}}+\phi_{2}\left(\sigma_{\mathrm{nf}}-\sigma_{n2}\right)},\frac{\sigma_{\mathrm{nf}}}{\sigma_{f}}=\frac{\sigma_{n1}+2\sigma_{f}-2\phi_{1}\left(\sigma_{f}-\sigma_{n1}\right)}{\sigma_{n}+2\sigma_{f}+\phi \left(\sigma_{f}-\sigma_{n1}\right)}, \end{array}$$

where $\mu_{f},\phi_{1},\phi_{2},\rho_{f},\kappa ,{\left(\mathrm{\rho Cp}\right)}_{f},\sigma_{\mathrm{hnf}},\mathrm{hnf},\mathrm{nf},f$ , and n the base fluid viscosity, nanoparticle volume fraction of Cu, nanoparticle volume fraction of ${\mathrm{Al}}_{2}O_{3}$ , density, thermal conductivity, heat capacitance, electrical conductivity of hybrid nanofluid, hybrid nanofluid, nanofluid, base fluid, and nanoparticle. Furthermore, in terms of shape, only spherical nanoparticles are taken into consideration. This hybrid nanofluid is applicable in aerospace, nuclear reactors, biomedical engineering, electronic cooling, and renewable energy systems, making it an optimal choice for advanced heat transfer applications.

Using the nonlinear Rosseland diffusion approximation, the radiative heat flux $q_{r}$ can be expressed as: ^46^

$$q_{r}=\frac{-4\sigma^{*}}{3\kappa^{*}}\frac{\partial T^{4}}{\partial z},$$ (16)

$$q_{r}=\frac{-16\sigma^{*}}{3\kappa^{*}}T^{3}\frac{\partial T}{\partial z},$$ (17)

and therefore

$$\frac{\partial q_{r}}{\partial z}=\frac{-16\sigma^{*}}{3\kappa^{*}}\frac{\partial }{\partial z}\left(T^{3}\frac{\partial T}{\partial z}\right).$$ (18)

By means of Equations (16), (17) and (18), Equation (13) reduces to

$$u\frac{\partial T}{\partial x}+v\frac{\partial T}{\partial y}+w\frac{\partial T}{\partial z}=\alpha_{\mathrm{hnf}}\frac{\partial^{2}T}{\partial z^{2}}+\frac{1}{{\left(\mathrm{\rho Cp}\right)}_{\mathrm{hnf}}}\frac{16\sigma^{*}}{3\kappa^{*}}\frac{\partial }{\partial z}\left(T^{3}\frac{\partial T}{\partial z}\right)-\lambda_{e}[u^{2}\frac{\partial^{2}T}{\partial x^{2}}+v^{2}\frac{\partial^{2}T}{\partial y^{2}}+w^{2}\frac{\partial^{2}T}{\partial z^{2}}+2\left(\mathrm{uv}\frac{\partial^{2}T}{\partial x\partial y}+\mathrm{vw}\frac{\partial^{2}T}{\partial y\partial z}+\mathrm{uw}\frac{\partial^{2}T}{\partial x\partial z}\right)+\left(u\frac{\partial u}{\partial x}+v\frac{\partial u}{\partial y}+w\frac{\partial u}{\partial z}\right)\frac{\partial T}{\partial x}+\left(u\frac{\partial v}{\partial x}+v\frac{\partial v}{\partial y}+w\frac{\partial v}{\partial z}\right)\frac{\partial T}{\partial y}+\left(u\frac{\partial w}{\partial x}+v\frac{\partial w}{\partial y}+w\frac{\partial w}{\partial z}\right)\frac{\partial T}{\partial z}]+\frac{Q_{0}\left(T-T_{\infty }\right)}{{\left(\mathrm{\rho Cp}\right)}_{\mathrm{hnf}}}+\frac{\sigma_{\mathrm{hnf}}B^{2}\left(u^{2}+v^{2}\right)}{{\left(\mathrm{\rho Cp}\right)}_{\mathrm{hnf}}}+\tau \left[D_{B}\frac{\partial C}{\partial z}\frac{\partial T}{\partial z}+\frac{D_{T}}{T_{\infty }}{\left(\frac{\partial^{2}T}{\partial z^{2}}\right)}^{2}\right].$$ (19)

The similarity transformations are given by ^53, 55^

$$\left\{ \begin{array}{l} w=-\sqrt{a\nu_{f}}\left(f\left(\eta \right)+g\left(\eta \right)\right),u=\mathrm{ax}f^{\prime }\left(\eta \right),v=\mathrm{ay}g^{\prime }\left(\eta \right),\eta =z\sqrt{\frac{a}{\nu_{f}}}\\ T=T_{\infty }+\left(T_{w}-T_{\infty }\right)\theta \left(\eta \right),C=C_{\infty }+\left(C_{w}-C_{\infty }\right)\Phi \left(\eta \right). \end{array}\right.$$ (20)

Equations (10) to (14) are simplified accordingly:

$$\frac{A_{1}}{A_{2}}f^{\prime \prime \prime }\left[1+\text{We}f^{\prime \prime }\right]+\left(f+g\right)f^{\prime \prime }-f^{\prime 2}-\frac{A_{3}}{A_{2}}Mf^{\prime }-\frac{A_{1}}{A_{2}}Kf^{\prime }=0,$$ (21)

$$\frac{A_{1}}{A_{2}}g^{\prime \prime \prime }\left[1+\text{We}g^{\prime \prime }\right]+\left(f+g\right)g^{\prime \prime }-g^{\prime 2}-\frac{A_{3}}{A_{2}}Mg^{\prime }-\frac{A_{1}}{A_{2}}Kg^{\prime }=0,$$ (22)

$$\begin{array}{c} \frac{A_{4}}{A_{5}\mathrm{Pr}}\theta^{\prime \prime }+\left(f+g\right)\theta^{\prime }-\beta_{1}\left[{\left(f+g\right)}^{2}\theta^{\prime \prime }+\left(f+g\right)\left(f^{\prime }+g^{\prime }\right)\theta^{\prime }\right]+\\ \frac{1}{A_{5}\mathrm{Pr}}R\left[{\left(1+\left(\theta_{w}-1\right)\right)}^{2}\times 3\left(\theta_{w}-1\right)\theta^{\prime 2}+{\left(1+\left(\theta_{w}-1\right)\theta \right)}^{3}\theta^{\prime \prime }\right]+\\ \frac{1}{A_{5}}\mathrm{Q\theta}+\mathrm{Nb}\theta^{\prime }\Phi^{\prime }+\mathrm{Nt}\theta^{2}+\frac{A_{3}}{A_{5}}M\left(\mathrm{Ec}{\left(f^{\prime }\right)}^{2}+\mathrm{Ec}{\left(g^{\prime }\right)}^{2}\right)=0, \end{array}$$ (23)

$$\Phi^{\prime \prime }+\frac{\mathrm{Nt}}{\mathrm{Nb}}\theta^{\prime \prime }+\mathrm{Sc}\left(f+g\right)\Phi^{\prime }-\beta_{2}\mathrm{Sc}\left[{\left(f+g\right)}^{2}\Phi^{\prime \prime }+\left(f+g\right)\left(f^{\prime }+g^{\prime }\right)\Phi^{\prime }\right]-\mathrm{ScH}\Phi =0.$$ (24)

with subject to boundary conditions

$$\left\{ \begin{array}{l} f^{\prime }\left(0\right)=1,f\left(0\right)=0,g\left(0\right)=0,g^{\prime }\left(0\right)=d,\theta \left(0\right)=1,\Phi \left(0\right)=1,\mathrm{at}\;\eta =0\\ f^{\prime }\left(\eta \right)=0,g^{\prime }\left(\eta \right)=0,\theta \left(\eta \right)=0,\Phi \left(\eta \right)=0,\mathrm{as}\;\eta \to \infty \end{array}\right.$$ (25)

where, the Weissenberg number (We) , magnetic field parameter (M) , porosity parameter (K) , Prandtl number (Pr) , non-linear thermal radiation (R) , ratio of temperature $\left(\theta_{w}\right),Q$ is the heat generation (Q>0) or absorption parameter (Q<0) , Brownian motion parameter (Nb) , Thermophoresis parameter (Nt) , Eckert number (Ec) along the x - and y -axis, thermal relaxation parameter $\left(\beta_{1}\right)$ , concentration relaxation parameter $\left(\beta_{2}\right)$ , Schmidt number (Sc) , Chemical reaction (H),d is ratio of stretching sheet, and $A_{1},A_{2},A_{3},A_{4}$ , and $A_{5}$ are dynamic viscosity, density, electrical conductivity, thermal conductivity, and heat capacitance of the hybrid nanofluid, respectively can be described as follows:

$$\begin{array}{c} \text{We}=x\Gamma \sqrt{\frac{2a^{3}}{\nu_{f}}},M=\frac{\sigma_{f}B^{2}}{\rho_{f}a},K=\frac{\nu_{f}}{aK_{p}},\mathrm{Pr}=\frac{\nu_{f}{\left(\mathrm{\rho Cp}\right)}_{f}}{\kappa_{f}},R=\frac{16\sigma^{*}T_{\infty }^{3}}{3\kappa \kappa^{*}},\theta_{w}=\frac{T_{w}}{T_{\infty }}\\ Q=\frac{Q_{0}}{a{\left(\mathrm{\rho Cp}\right)}_{f}},\mathrm{Nb}=\frac{D_{B}\tau \left(C_{w}-C_{\infty }\right)}{\nu_{f}},\mathrm{Nt}=\frac{D_{T}\tau \left(T_{w}-T_{\infty }\right)}{\nu_{f}T_{\infty }},Ec_{x}=\frac{u_{w}^{2}}{\left(T_{w}-T_{\infty }\right){\left(\mathrm{Cp}\right)}_{f}}\\ Ec_{y}=\frac{v_{w}^{2}}{\left(T_{w}-T_{\infty }\right){\left(C_{p}\right)}_{f}},\beta_{1}=\lambda_{e}a,\beta_{2}=\lambda_{c}a,\mathrm{Sc}=\frac{\nu_{f}}{D_{B}},H=\frac{K_{r}}{a},d=\frac{b}{a}\\ A_{1}=\frac{\mu_{\mathrm{hnf}}}{\mu_{f}},A_{2}=\frac{\rho_{\mathrm{hnf}}}{\rho_{f}},A_{3}=\frac{\sigma_{\mathrm{hnf}}}{\sigma_{f}},A_{4}=\frac{\kappa_{\mathrm{hnf}}}{\kappa_{f}},A_{5}=\frac{{\left(\mathrm{\rho Cp}\right)}_{\mathrm{hnf}}}{{\left(\mathrm{\rho Cp}\right)}_{f}}. \end{array}$$

The engineering components of the skin friction coefficients $C_{\mathrm{fx}}$ and $C_{\mathrm{fy}}$ , Nusselt number $Nu_{x}$ and Sherwood number $Sh_{x}$ are defined as

$$C_{\mathrm{fx}}=\frac{\tau_{\mathrm{xz}}}{\rho_{f}u_{w}^{2}},C_{\mathrm{fy}}=\frac{\tau_{\mathrm{yz}}}{\rho_{f}v_{w}^{2}},Nu_{x}=\frac{xq_{w}}{\kappa_{f}\left(T_{w}-T_{\infty }\right)},Sh_{x}=\frac{xj_{m}}{D_{B}\left(C_{w}-C_{\infty }\right)},$$ (26)

where $\tau_{\mathrm{xz}}$ and $\tau_{\mathrm{yz}}$ are the wall shear stresses in the x- and y-direction respectively, given as

$$\tau_{\mathrm{xz}}=\mu_{\mathrm{hnf}}{\left[\frac{\partial u}{\partial z}+\frac{\Gamma }{\sqrt{2}}{\left(\frac{\partial u}{\partial z}\right)}^{2}\right]}_{z=0},\tau_{\mathrm{yz}}=\mu_{\mathrm{hnf}}{\left[\frac{\partial v}{\partial z}+\frac{\Gamma }{\sqrt{2}}{\left(\frac{\partial v}{\partial z}\right)}^{2}\right]}_{z=0}$$ (27)

and the wall heat and mass flux from the sheets, which is given by:

$$q_{w}=-\left[\kappa_{\mathrm{hnf}}+\frac{16\sigma^{*}T_{\infty }^{3}}{3\kappa^{*}}\right]{\left(\frac{\partial T}{\partial z}\right)}_{z=0},j_{m}=-D_{B}{\left(\frac{\partial C}{\partial z}\right)}_{z=0}.$$ (28)

By using Equations (26), (27) and (28), the dimensionless variables, we obtain:

$${\left(Re_{x}\right)}^{\frac{1}{2}}C_{\mathrm{fx}}=A_{1}\left[1+\frac{\text{We}}{2}f^{\prime \prime }\left(0\right)\right]f^{\prime \prime }\left(0\right),$$ (29)

$$d^{1.5}{\left(Re_{y}\right)}^{\frac{1}{2}}C_{\mathrm{fy}}=A_{1}\left[1+\frac{\text{We}}{2}g^{\prime \prime }\left(0\right)\right]g^{\prime \prime }\left(0\right),$$ (30)

$${\left(Re_{y}\right)}^{-\frac{1}{2}}Nu_{x}=-\left(A_{4}+R\right)\theta^{\prime }\left(0\right),$$ (31)

$${\left(Re_{y}\right)}^{-\frac{1}{2}}Sh_{x}=-\Phi^{\prime }\left(0\right),$$ (32)

where $Re_{y}=\frac{yv_{w}}{\nu_{f}}$ and $Re_{x}=\frac{xu_{w}}{\nu_{f}}$ are the local Reynolds numbers.

## 3. Numerical method

Using Table 1, and the MATLAB software ( https://github.com/asfawmat/BVP-MATLAB-Implementation) uses the bvp4c technique to numerically solve equations (21) through (24) and the boundary condition (25). The bvp4c is an effective instrument that provides precision and dependability in addressing boundary value problems for differential equation systems. BVP4C is chosen for its high accuracy, adaptive mesh refinement, and robustness in handling boundary value problems (BVPs), making it well-suited for nonlinear and coupled ODEs arising in Williamson hybrid nanofluid flow. Unlike shooting methods, BVP4C provides stability and effectively handles multi-point boundary conditions, making it ideal for fluid flow and heat transfer problems. Although methods like Runge-Kutta and finite differences were considered, they proved less efficient due to stability and convergence issues. However, BVP4C requires careful selection of initial guesses due to its computational intensity. With a high degree of accuracy specifically, fourth-order accuracy this solver exemplifies a collocation technique that provides a precise, continuous solution. The mesh selection and error control are determined by evaluating the residual of the continuous solution. As discussed later, the boundary value problems must be transformed into a system of first-order initial value problems (IVPs) to apply the bvp4c method using the shooting technique. Now let us define the new variable by the equation

$$\left\{ \begin{array}{l} y_{1}=f,y_{2}=f^{\prime },y_{3}=f^{\prime \prime },y_{4}=g,y_{5}=g^{\prime },\\ y_{6}=g^{\prime \prime },y_{7}=\theta ,y_{8}=\theta^{\prime },y_{9}=\Phi ,y_{10}=\Phi^{\prime }. \end{array}\right.$$ (33)

$$\begin{array}{c} y_{1}^{\prime }=y_{2}\\ y_{2}^{\prime }=y_{3}\\ y_{3}^{\prime }=\frac{1}{\frac{A_{1}}{A_{2}}\left(1+\text{We}y_{3}\right)}\left[\frac{A_{3}}{A_{2}}My_{2}+\frac{A_{1}}{A_{2}}Ky_{2}+y_{2}^{2}-\left(y_{1}+y_{4}\right)y_{3}\right],\\ y_{4}^{\prime }=y_{5},\\ y_{5}^{\prime }=y_{6},\\ y_{6}^{\prime }=\frac{1}{\frac{A_{1}}{A_{2}}\left(1+\text{We}y_{6}\right)}\left[\frac{A_{3}}{A_{2}}My_{5}+\frac{A_{1}}{A_{2}}Ky_{5}+y_{5}^{2}-\left(y_{1}+y_{4}\right)y_{6}\right],\\ y_{7}^{\prime }=y_{8},\\ y_{8}^{\prime }=-\left[\frac{1}{\left(\frac{A_{4}}{A_{5}\mathrm{Pr}}+\frac{R}{A_{5}\mathrm{Pr}}{\left(1+\left(\theta_{w}-1\right)y_{7}\right)}^{3}-\beta_{1}{\left(y_{1}+y_{4}\right)}^{2}\right)}\right]\\ \times [\frac{R}{A_{5}\mathrm{Pr}}{\left(1+\left(\theta_{w}-1\right)\right)}^{2}\times 3\left(\theta_{w}-1\right)y_{8}^{2}+\left(y_{1}+y_{4}\right)y_{8}\\ -\beta_{1}\left(y_{1}+y_{4}\right)\left(y_{2}+y_{5}\right)y_{8}+\frac{1}{A_{5}}Qy_{7}+\mathrm{Nb}y_{8}y_{10}+\mathrm{Nt}y_{8}^{2}\\ +\frac{A_{3}}{A_{5}}M\left(Ec{\left(y_{2}\right)}^{2}+Ec{\left(y_{5}\right)}^{2}\right)],\\ y\prime_{9}=y_{10},\\ y_{10}^{\prime }=\left[\frac{1}{\left(1-\beta_{2}\mathrm{Sc}{\left(y_{1}+y_{4}\right)}^{2}\right)}\right]\times [\beta_{2}\mathrm{Sc}\left(\left(y_{1}+y_{4}\right)\left(y_{2}+y_{5}\right)-\mathrm{Sc}\left(y_{1}+y_{4}\right)\right)y_{10}\\ \;+\mathrm{ScH}y_{9}-\frac{\mathrm{Nt}}{\mathrm{Nb}}[-\left[\frac{1}{\left(\frac{A_{4}}{A_{5}\mathrm{Pr}}+\frac{R}{A_{5}\mathrm{Pr}}{\left(1+\left(\theta_{w}-1\right)\theta \right)}^{3}-\beta_{1}{\left(y_{1}+y_{4}\right)}^{2}\right)}\right]\\ \;\times [\frac{R}{A_{5}\mathrm{Pr}}\left({\left(1+\left(\theta_{w}-1\right)\right)}^{2}\times 3\left(\theta_{w}-1\right)y_{8}^{2}\right)+\left(y_{1}+y_{4}\right)y_{8}-\beta_{1}\left(y_{1}+y_{4}\right)\left(y_{2}+y_{5}\right)y_{8}\\ +\frac{1}{A_{5}}Qy_{7}+\mathrm{Nb}y_{8}y_{10}+\mathrm{Nt}y_{8}^{2}+\frac{A3}{A5}M\left(Ec{\left(y_{2}\right)}^{2}+Ec{\left(y_{5}\right)}^{2}\right)]] \end{array}$$

with corresponding initial conditions

$$\left\{ \begin{array}{l} y_{1}\left(0\right)=0,y_{2}\left(0\right)=1,y_{3}\left(0\right)=\alpha_{1},y_{4}\left(0\right)=0,y_{5}\left(0\right)=d\\ y_{6}\left(0\right)=\alpha_{2},y_{7}\left(0\right)=1,y_{8}\left(0\right)=\alpha_{3},y_{9}\left(0\right)=1,y_{10}\left(0\right)=\alpha_{4} \end{array}\right.$$ (34)

where $\alpha_{1},\alpha_{2},\alpha_{3}$ , and $\alpha_{4}$ are the missing initial conditions.

**Table 1.** Thermophysical properties. ^52, 56^

Physical properties	Ethylene glycol( *C*_2_ *H*_6_ *O*_2_)( *f* )	Copper ( *Cu*( $\phi_{1}$ ))	Alumina ( *Al*_2_ *O*_3_( $\phi_{2}$ ))
*ρ*	1115	8933	3970
*C _p_*	2430	385	765
*κ*	0.253	400	40
*σ*	0.107	5.96×10 ^7^	3.5×10 ^7^

Utilizing the bvp4c technique, a system of differential equations of the form $y^{\prime }=f\left(x,y\right)$ is integrated according to the specified boundary conditions. The bvp4c routine uses finite difference method with an achievable accuracy of about $10^{-7}$ .

## 4. Results and Discussion

In this section, we present the numerical solutions to the problem, considering various physical effects. The system of equations (21)- (24) is solved using the numerical bvp4c method, which satisfies the boundary conditions given by equation (25). Additionally, we conducted a comparative analysis of our numerical results with those from previous studies to verify the accuracy of our solution and evaluate its consistency with earlier findings. The comparison of our findings with those from earlier research is presented in Table 2. Comparing our findings with those of previous studies, the comparison’s results show generally excellent agreement. Using a variety of graphs, the impacts of various physical factors on temperature, concentration, velocity, mass transfer rate, surface drag coefficient, and the hybrid *Cu-Al *_2_ *O*_3_ */*ethylene glycol nanofluid phase and nanofluid *Al*_2_ *O*_3_ */*ethylene glycol phase are displayed. In each figure, a comparison of mono- and hybrid nanofluids is also provided. The numerical computations are discussed by keeping $d=0.5,\mathrm{Sc}=1,\mathrm{Nb}=\mathrm{Nt}=0.1,\text{We}=0.2,R=\theta_{w}=1.2,K=0.1,\mathrm{Pr}=1,\mathrm{Ec}=0.2,\beta_{1}=\beta_{2}=0.1,H=0.2,Q=0.1,M=1,0.01\leq \phi_{1}\leq 0.05,0.01\leq \phi_{2}\leq 0.05$ throughout the complete study.

**Table 2.** Comparison of *f*″ (0) and *g*″ (0).

*d*	Wang ^57^		You & Wang ^53^		Present	
	$f^{\prime \prime }$	$g^{\prime \prime }$	$f^{\prime \prime }$	$g^{\prime \prime }$	$f^{\prime \prime }$	$g^{\prime \prime }$
0	-1	0	-1	-	-1	0
0.25	-1.04881	-0.19456	-1.04881	-	-1.048813	-0.194565
0.5	-1.09310	-0.46521	-1.09310	-	-1.093096	-0.465205
0.75	-1.13449	-0.79462	-1.13449	-	-1.134486	-0.794619
1	-1.17372	-1.17372	-1.17372	-	-1.173721	-1.173721

An analysis of the differences between the numerical results of Wang, ^57^ You & Wang, ^53^ and the current study’s conclusions by calculating the values of f"(0) and g"(0) under the conditions of *Pr* = 1, *Nb* = 1 *×* 10 *−*14, when *We* = *M* = *K* = $\beta_{1}$ = $\beta_{2}$ = *R* = $\theta_{w}$ = *Q* = *Nt* = *Ec* = *Sc* = *H* = $\phi_{1}=\phi_{2}$ = 0, as shown in Table 2 below.

### 4.1 Velocity characteristics

**Figure 2.**
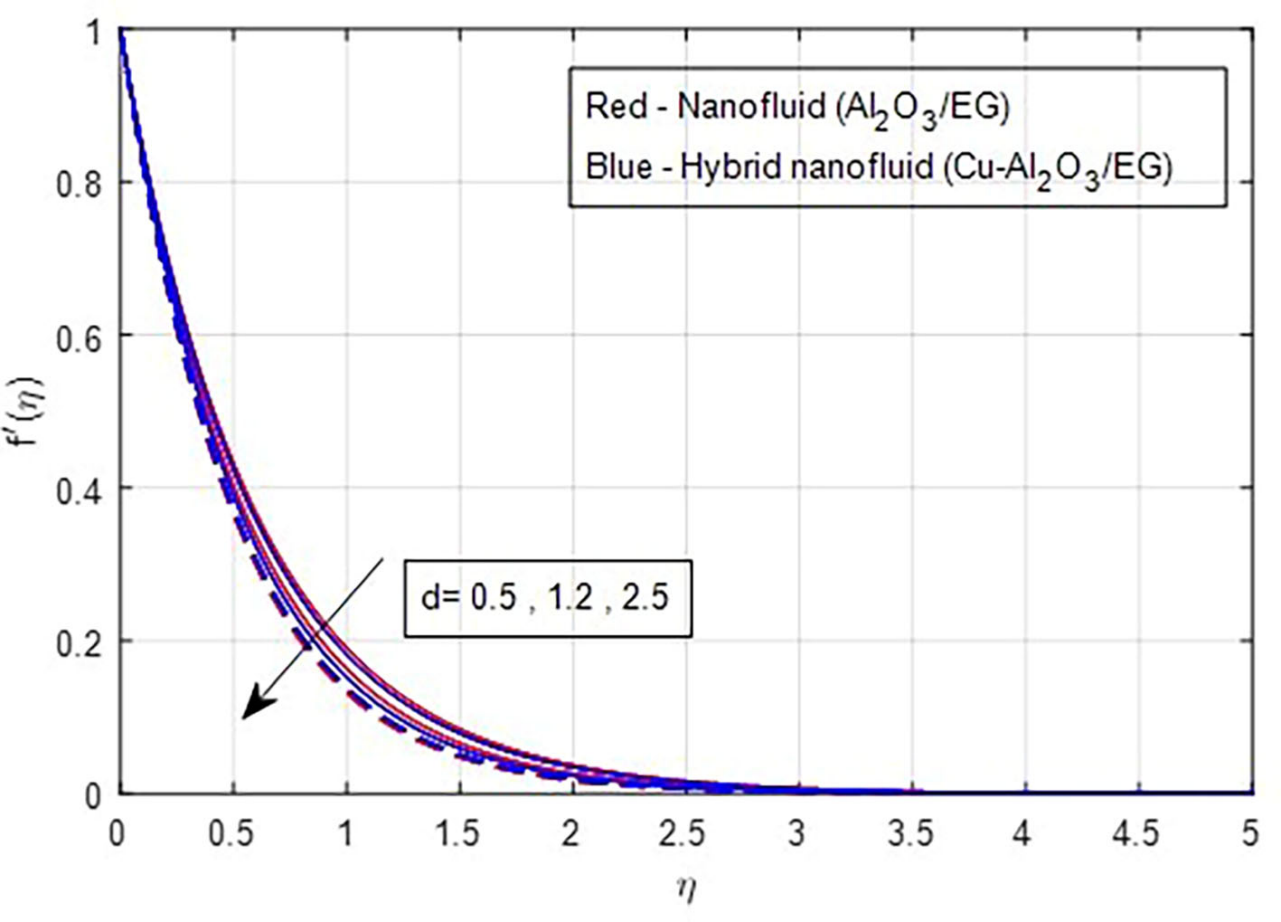
Primary velocity vs *d.*

**Figure 3.**
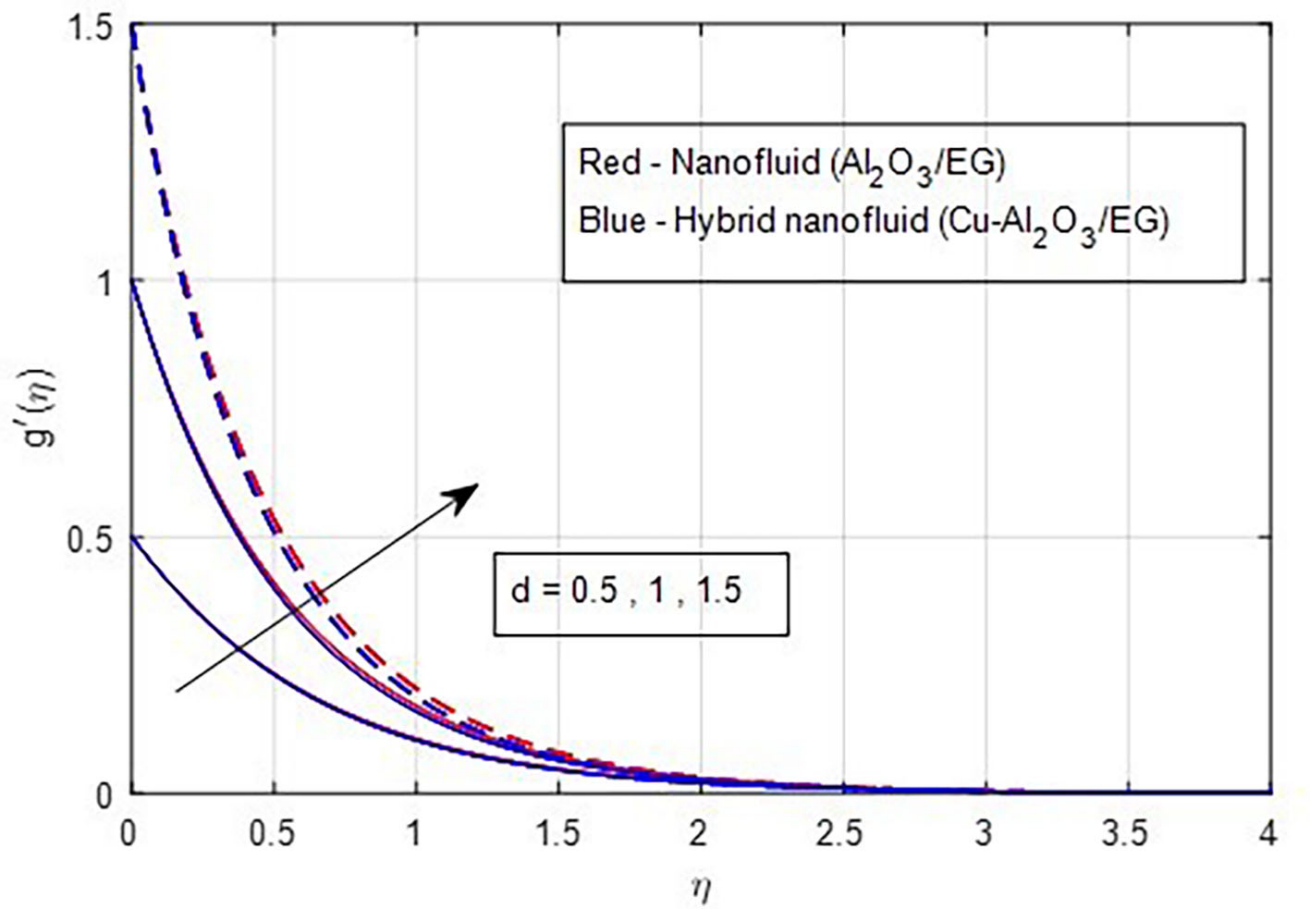
Secondary velocity vs *d.*

**Figure 4.**
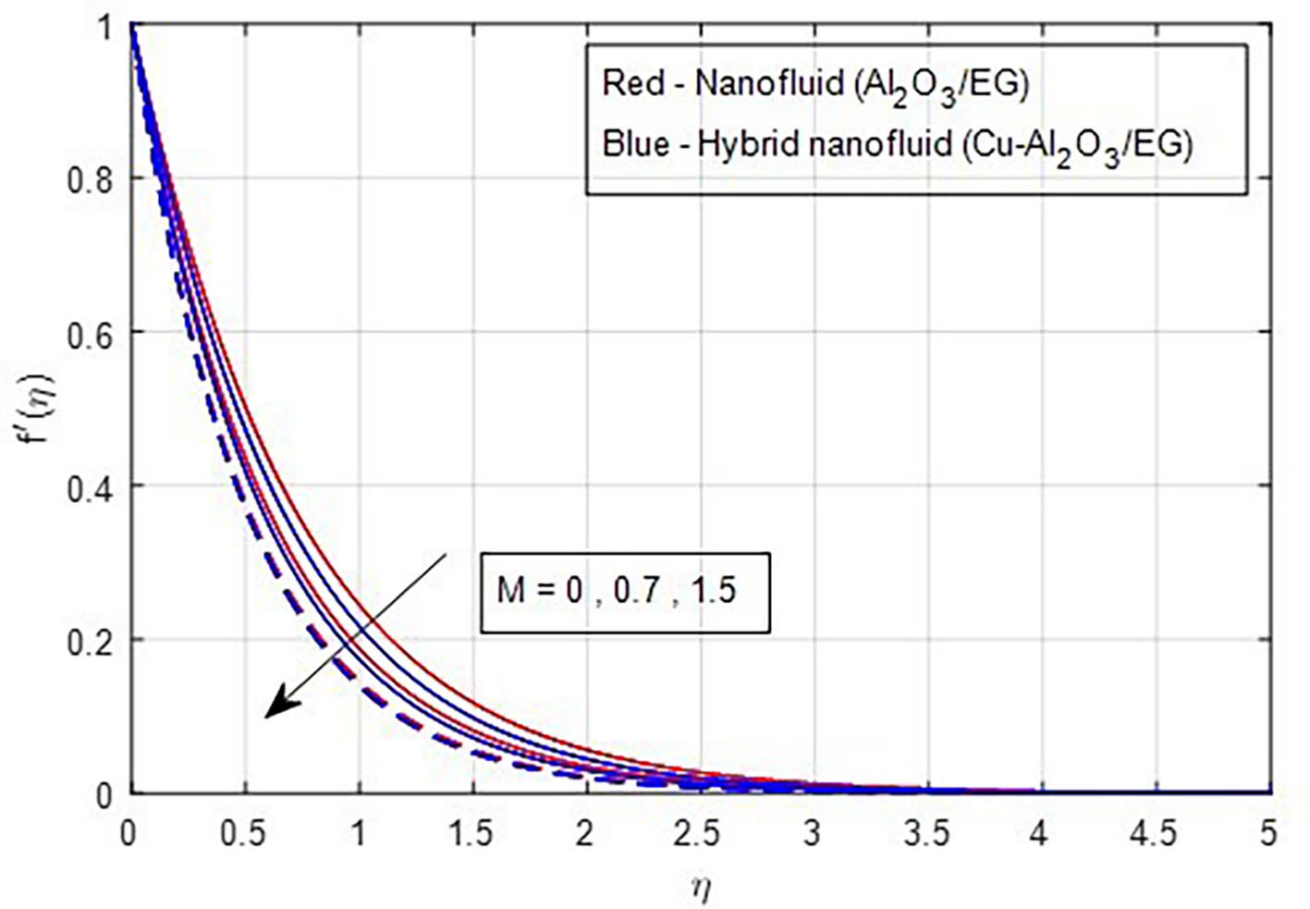
Primary velocity vs *M.*

**Figure 5.**
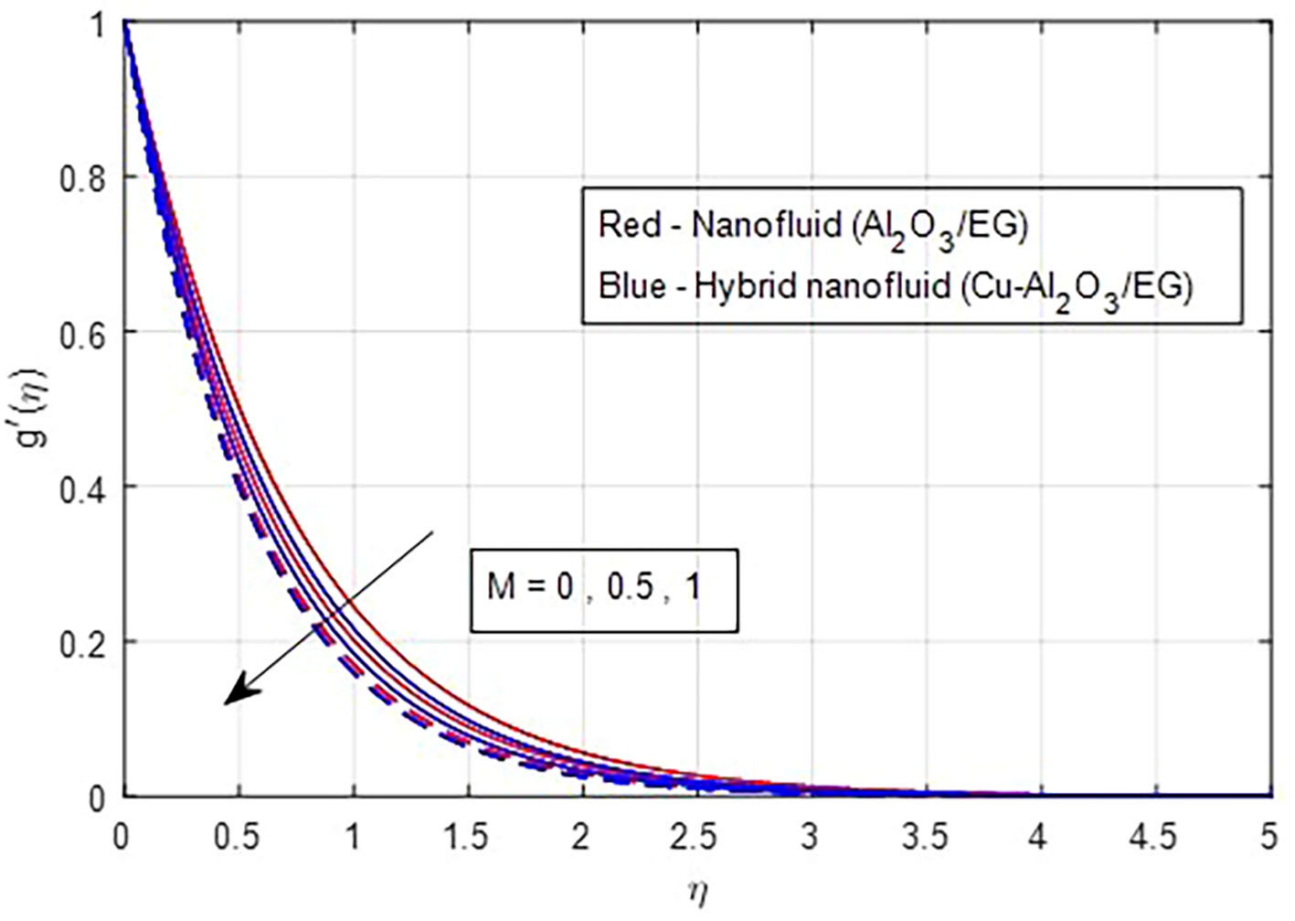
Secondary velocity vs *M*.

**Figure 6.**
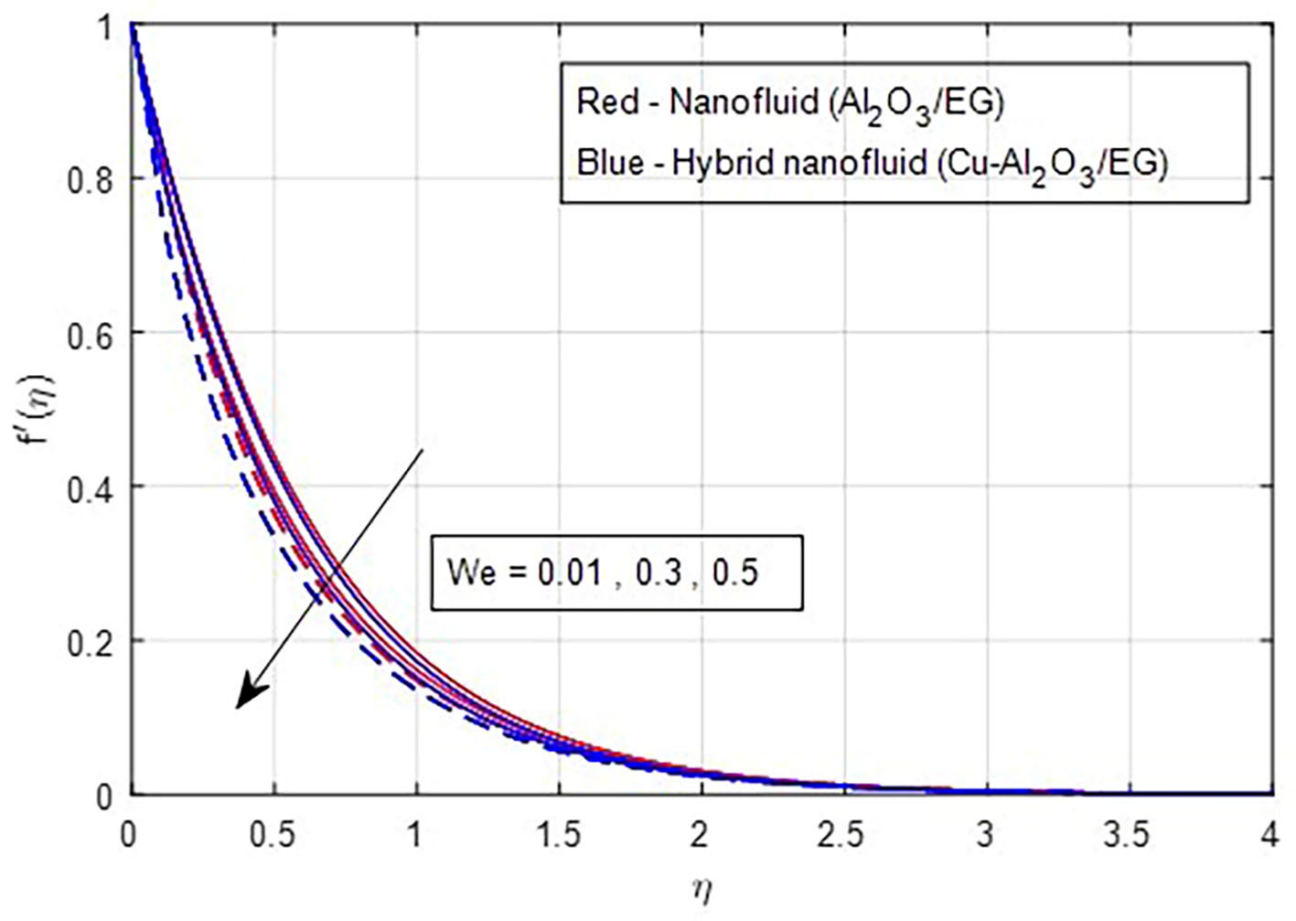
Primary velocity vs *We.*

**Figure 7.**
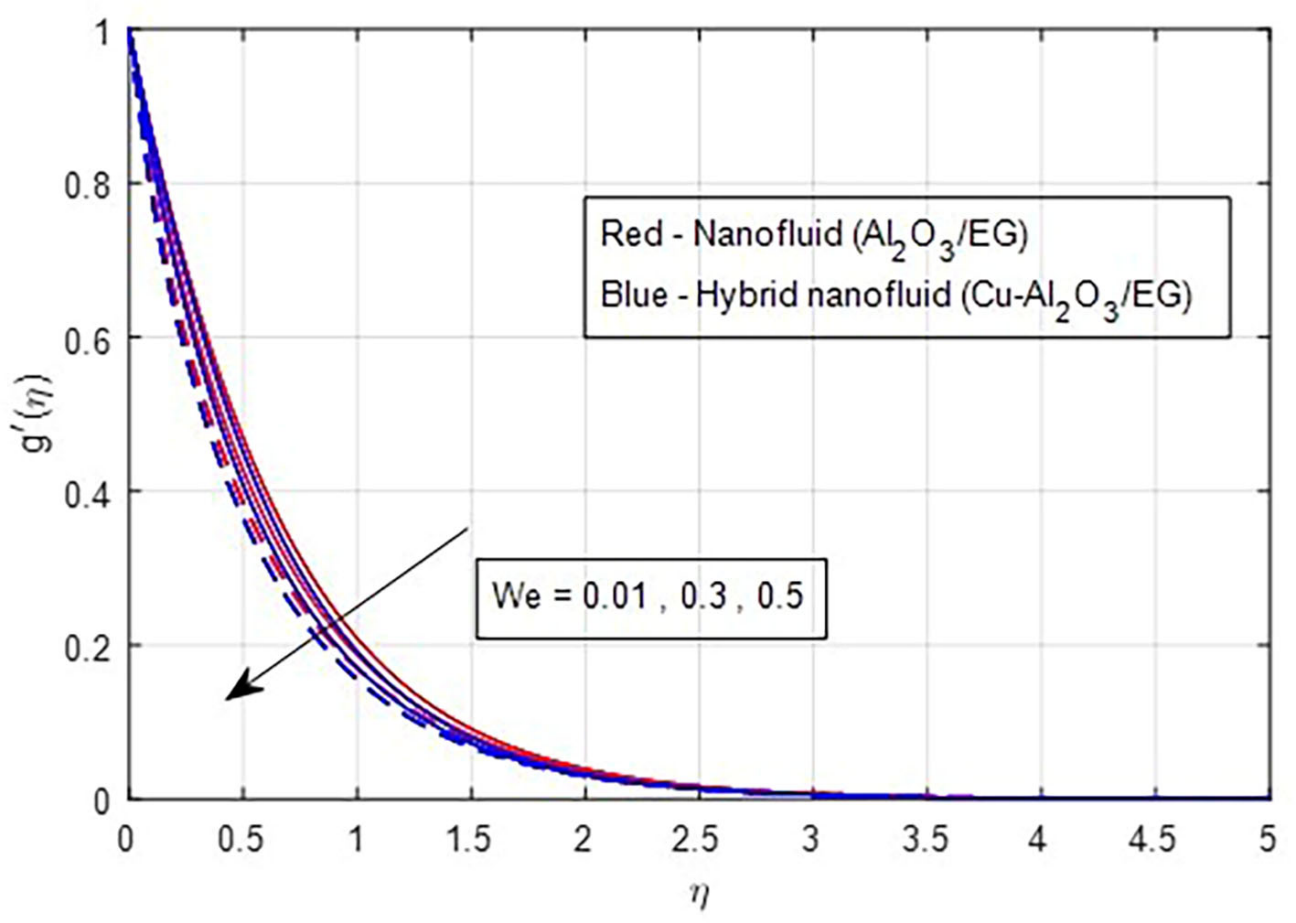
Secondary velocity vs *We.*

**Figure 8.**
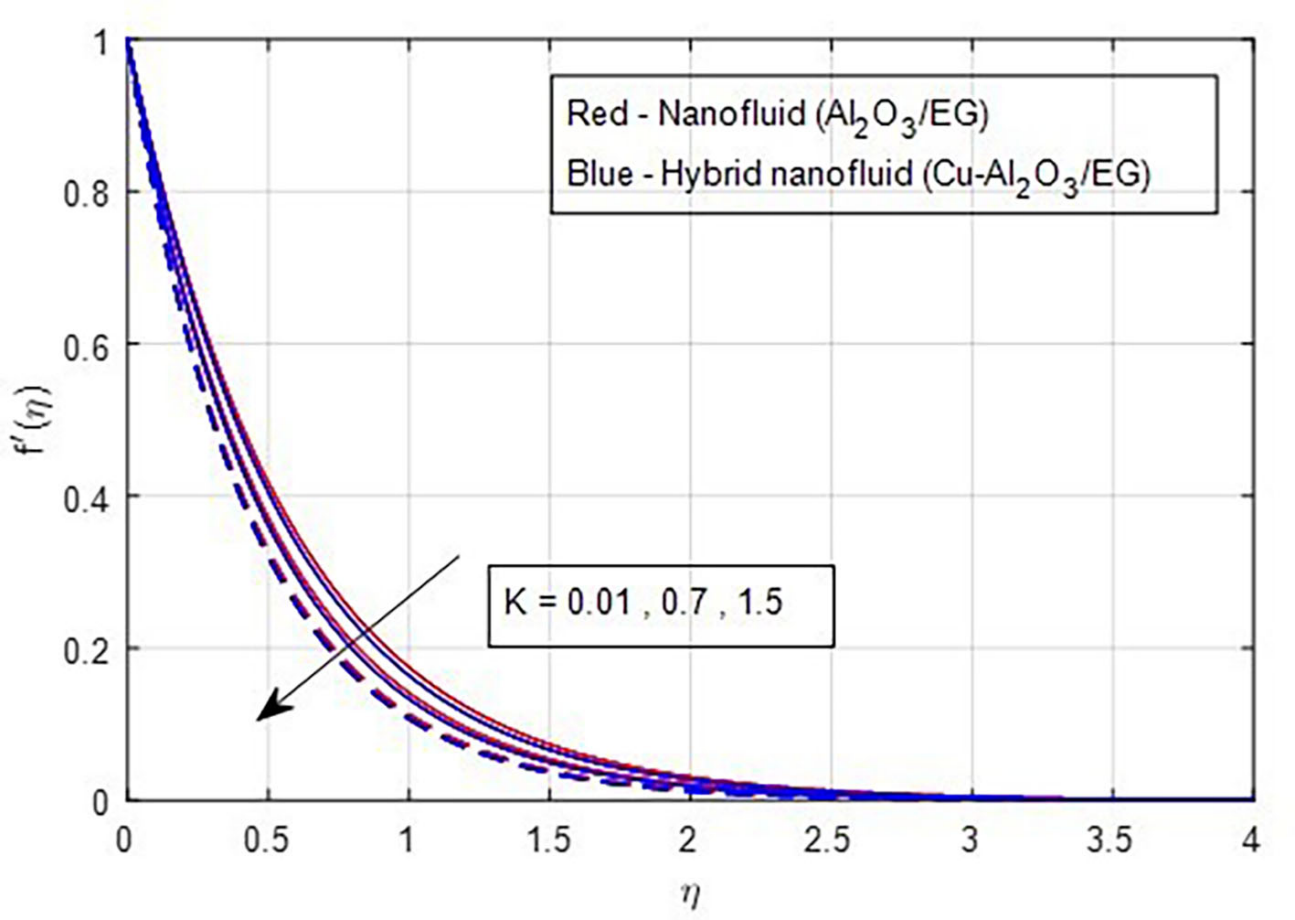
Primary velocity vs *K.*

**Figure 9.**
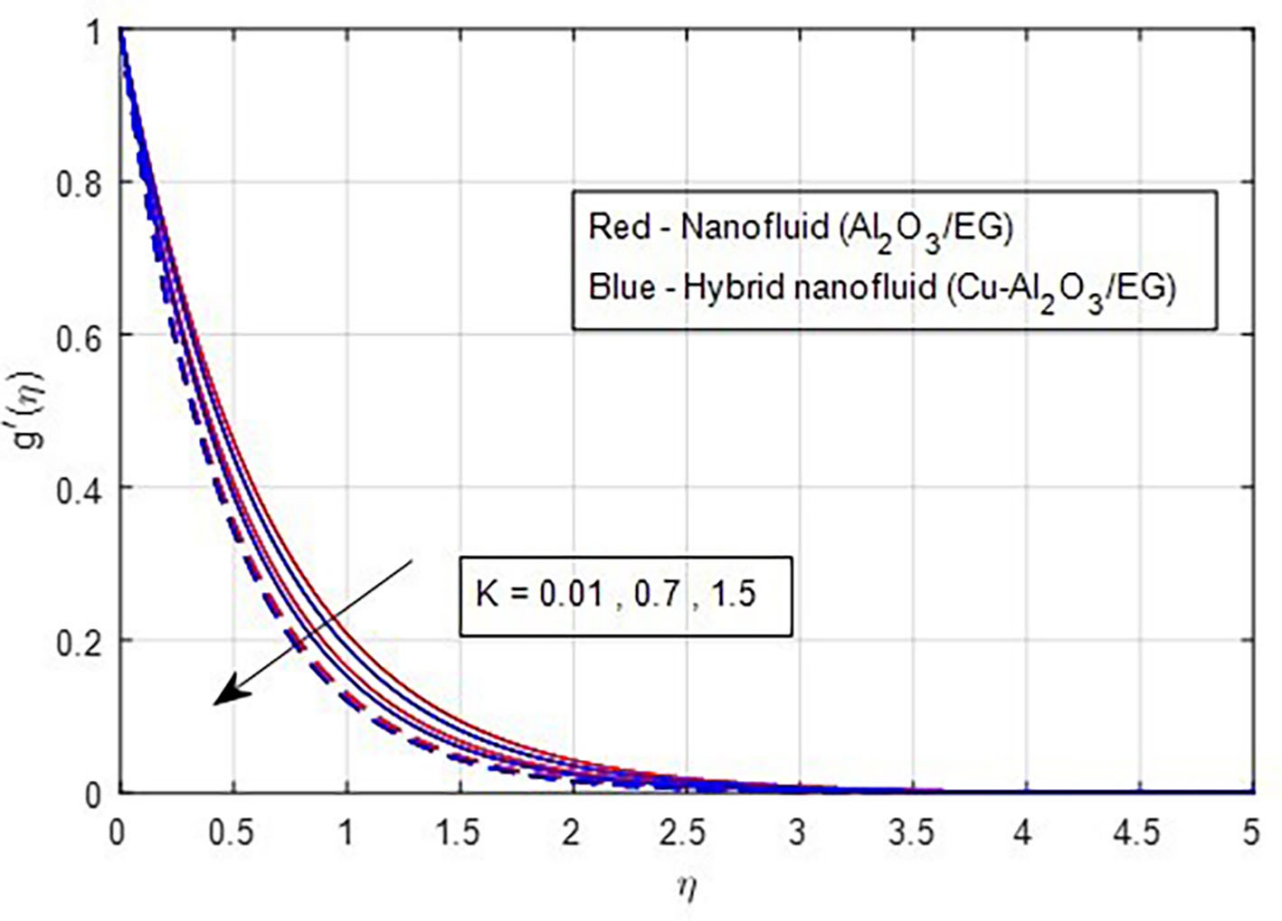
Secondary velocity vs *K.*

**Figure 10.**
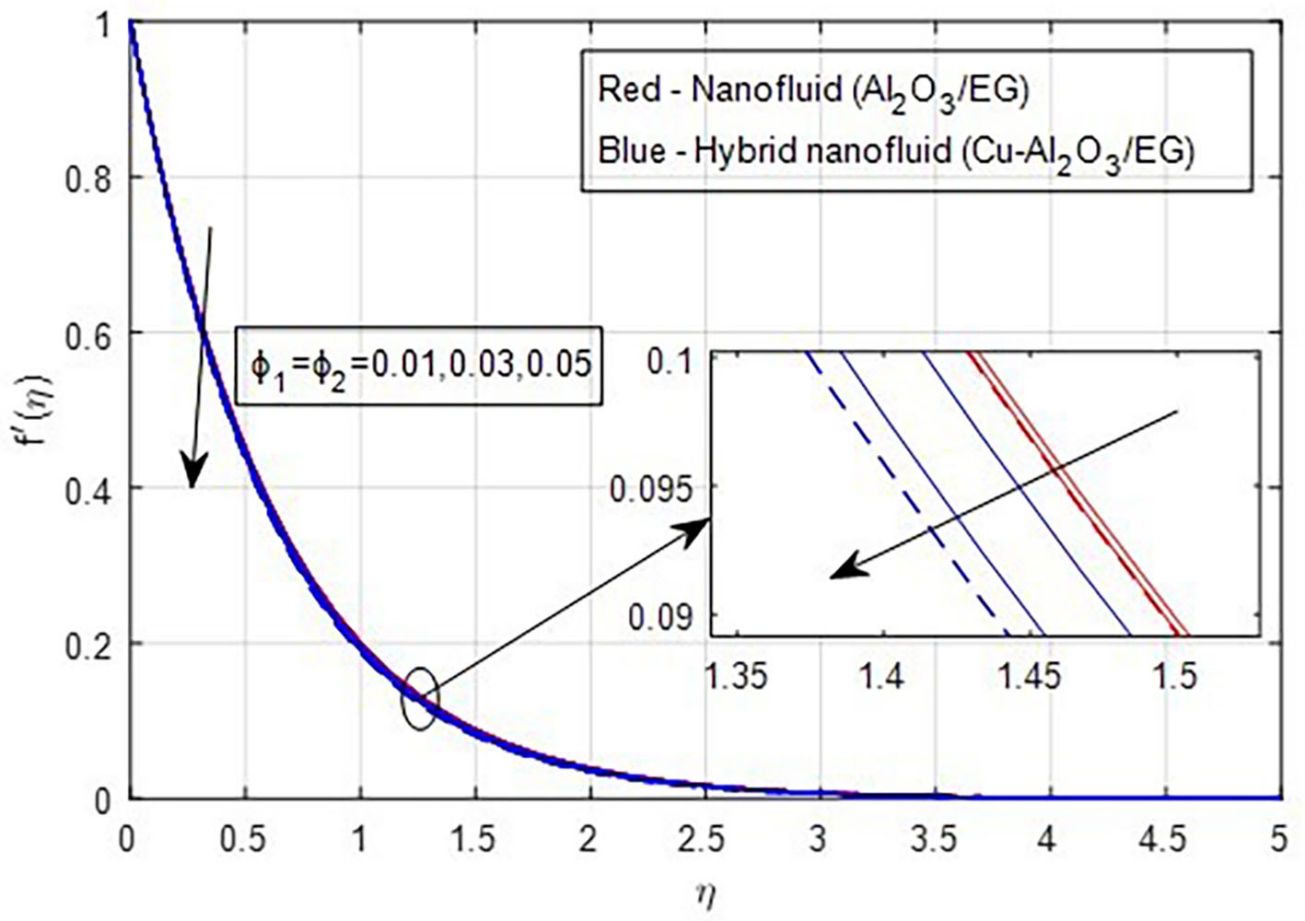
Primary velocity vs $\phi_{1},\phi_{2}$ .

**Figure 11.**
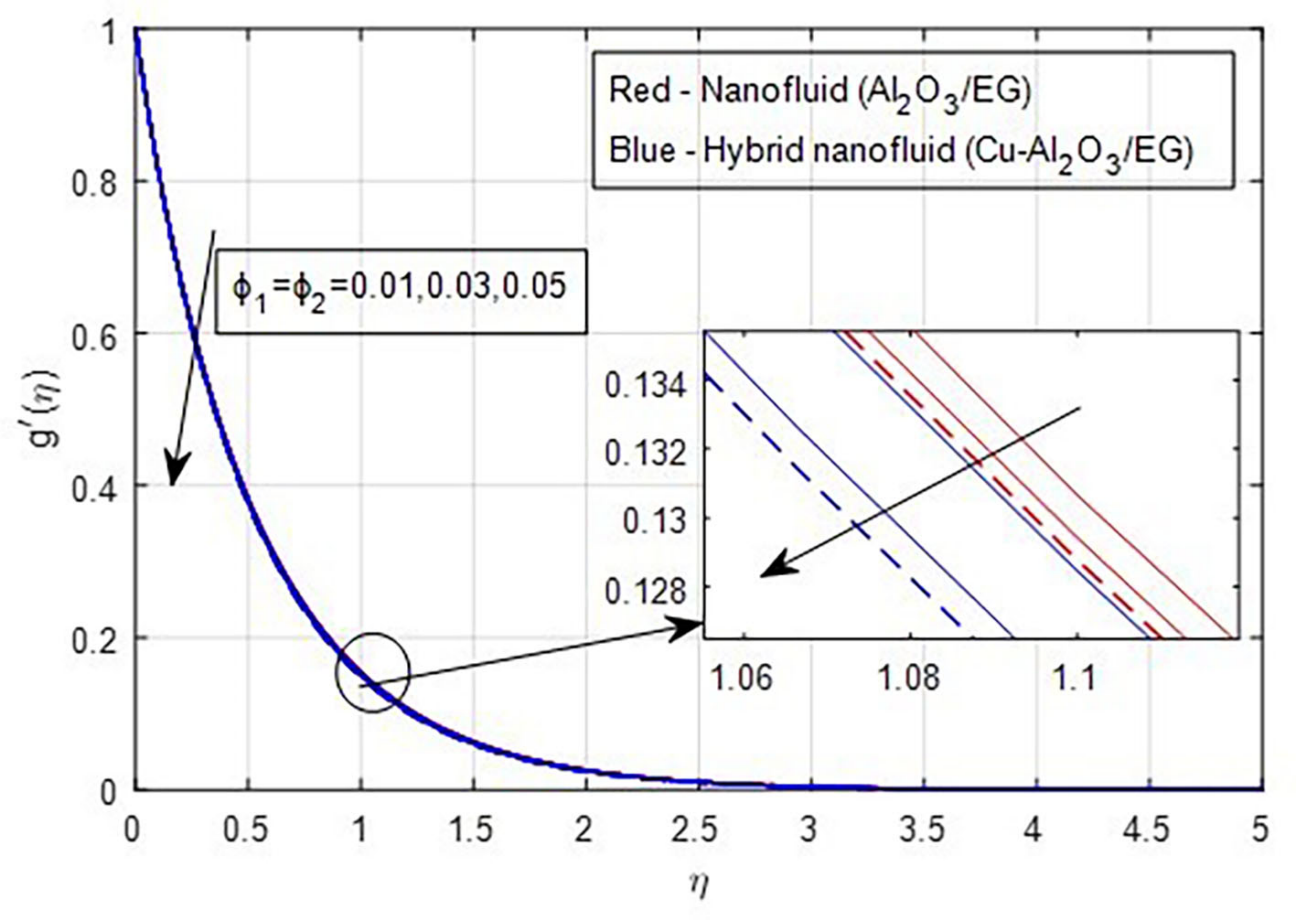
Secondary velocity vs $\phi_{1},\phi_{2}$ .

Figures 2 and 3 illustrate the impact of the stretching ratio on both primary and secondary velocities. As the stretching ratio increases, the primary velocity profiles decrease, while the secondary velocity profiles of the MHD Williamson hybrid nanofluid flow increase. Because d=b/a it decreases when the stretching rate is applied along the x-axis direction. It is evident that the velocity component (a) in the x-direction and the velocity component (b) in the y-direction have an inverse connection with the stretching ratio parameter. The interaction between primary and secondary velocity with a magnetic field is depicted in Figures 4 and 5. As the magnetic field strength increases, the resistance force also increases. The Lorentz force, which arises from the magnetic field, represents the resistance to fluid motion and can hinder the flow. As the magnetic field strengthens, both primary and secondary velocity profiles decrease. Figures 6 and 7 illustrate the effect of the Weissenberg number on these velocities. As the Weissenberg number increases, the velocity profiles (both primary and secondary) decrease because higher Weissenberg values reduce the relaxation time of the fluid particles. This increase in viscosity results in greater resistance to the fluid flow. Figures 8 and 9 demonstrate how the primary and secondary velocity profiles fall as the porosity parameter rises. This is because the hybrid nanofluid will pass through the sheet more readily as its permeability increases, influencing the flow. The contribution of volume fraction to the primary and secondary velocities of the mono- and hybrid nanofluids is shown in Figures 10 and 11. This variation demonstrates how increased volume friction will result in an increase in the fluid’s viscous forces, which are its internal resistive forces. When nanoparticles are added to ethylene glycol, the viscous forces in the fluid increase, and the fluid’s velocity decreases.

### 4.2 Thermal characteristics

**Figure 12.**
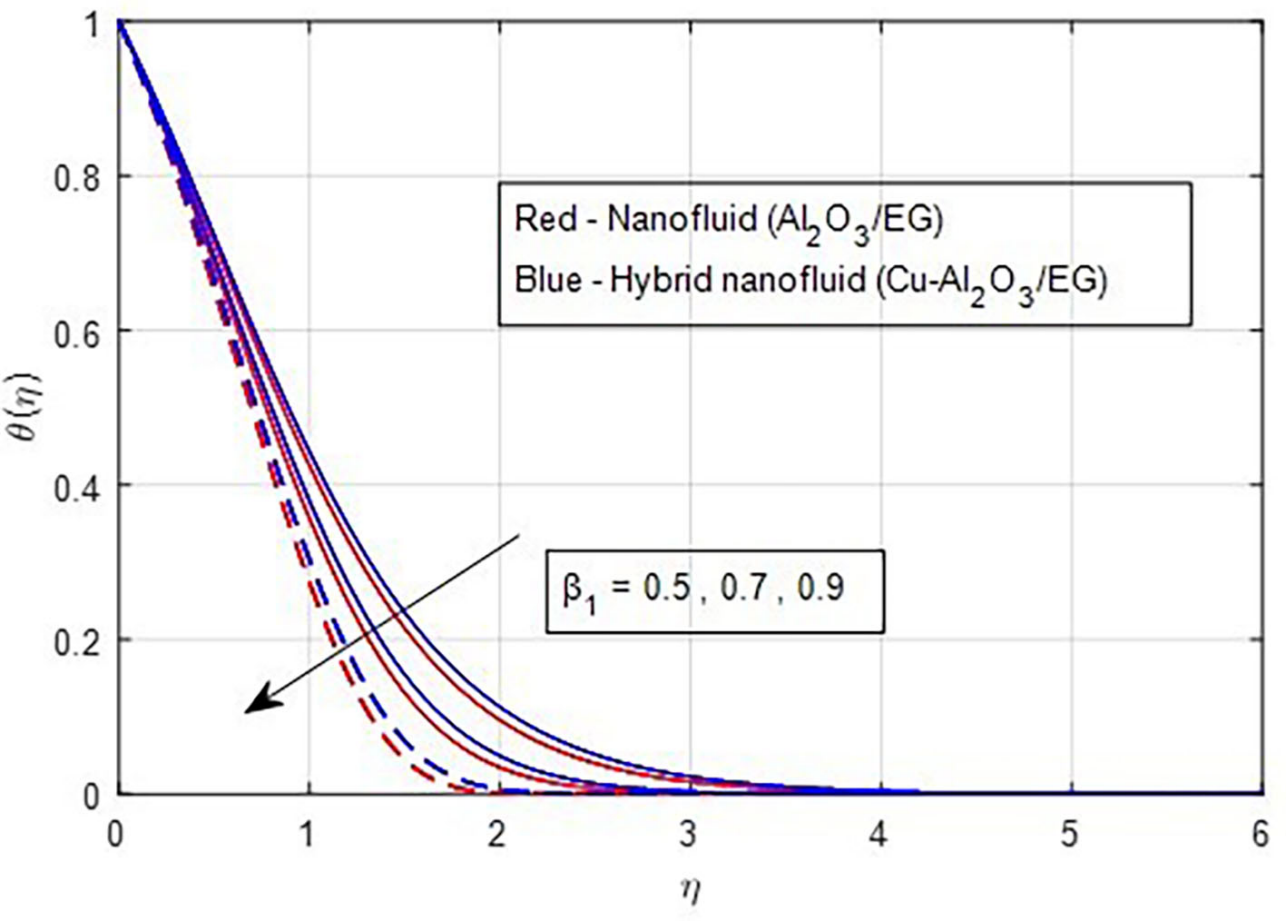
Temperature vs $\beta_{1}$ .

**Figure 13.**
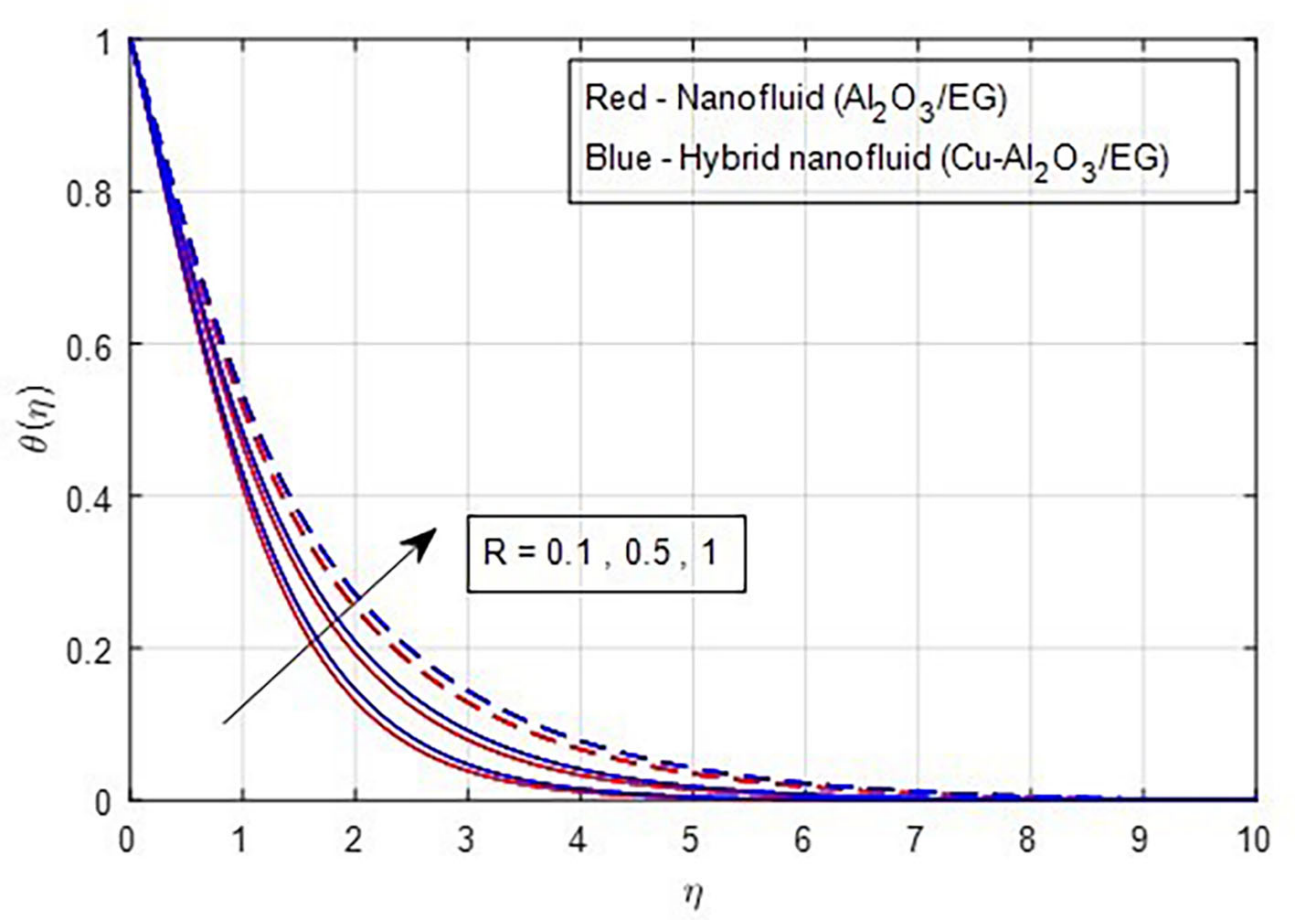
Temperature vs *R.*

**Figure 14.**
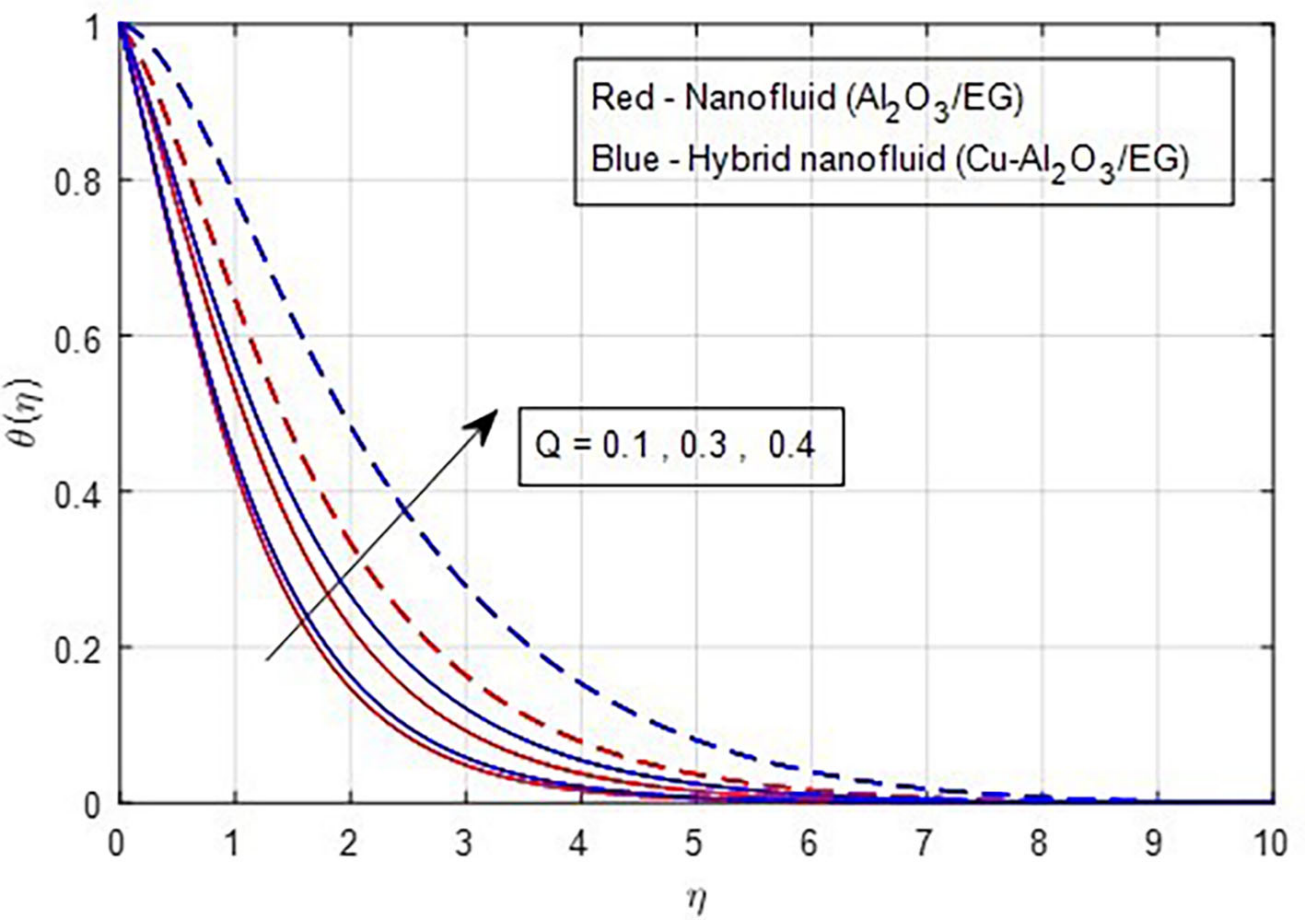
Temperature vs *Q.*

**Figure 15.**
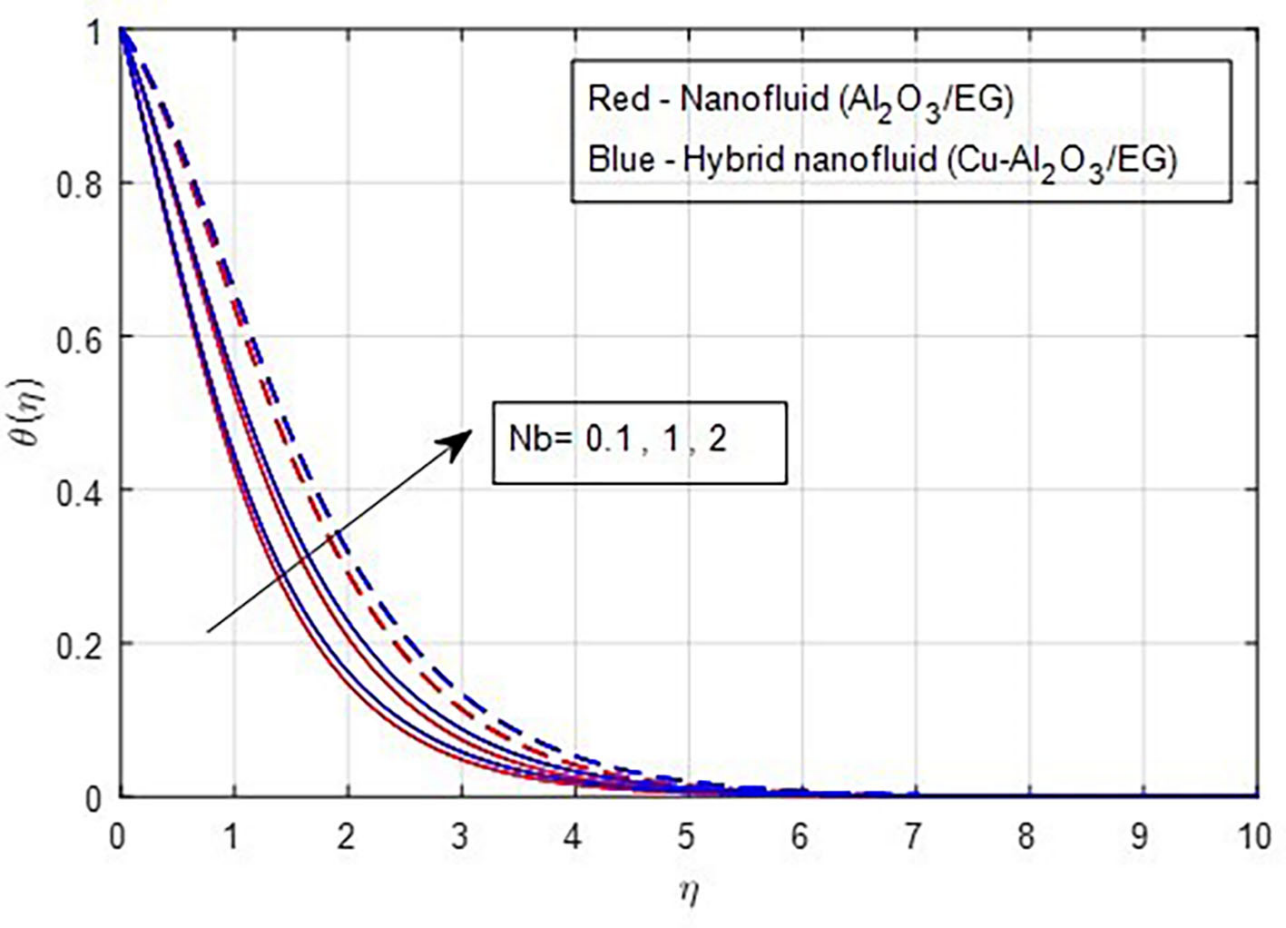
Temperature vs *Nb.*

**Figure 16.**
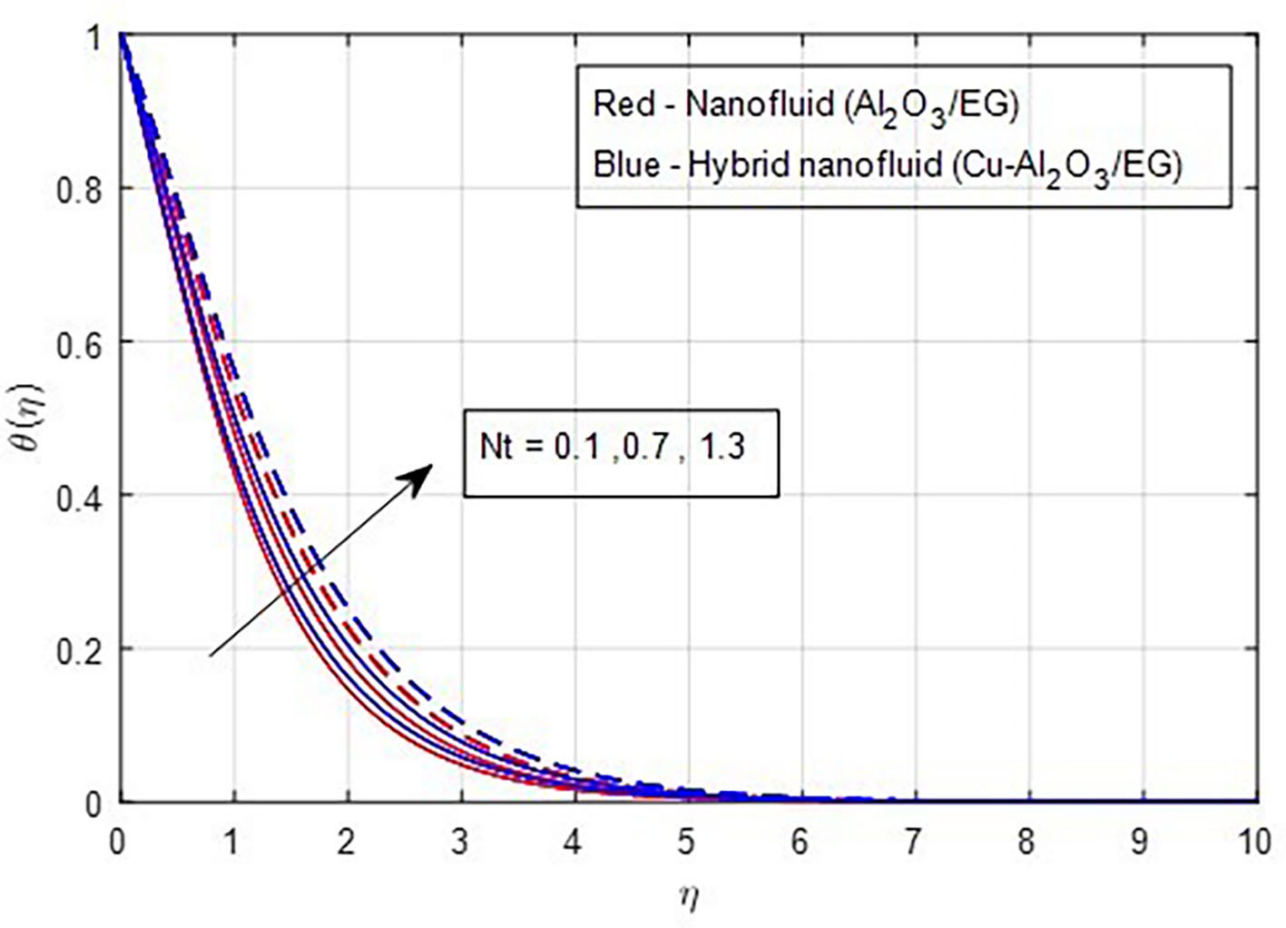
Temperature vs *Nt.*

**Figure 17.**
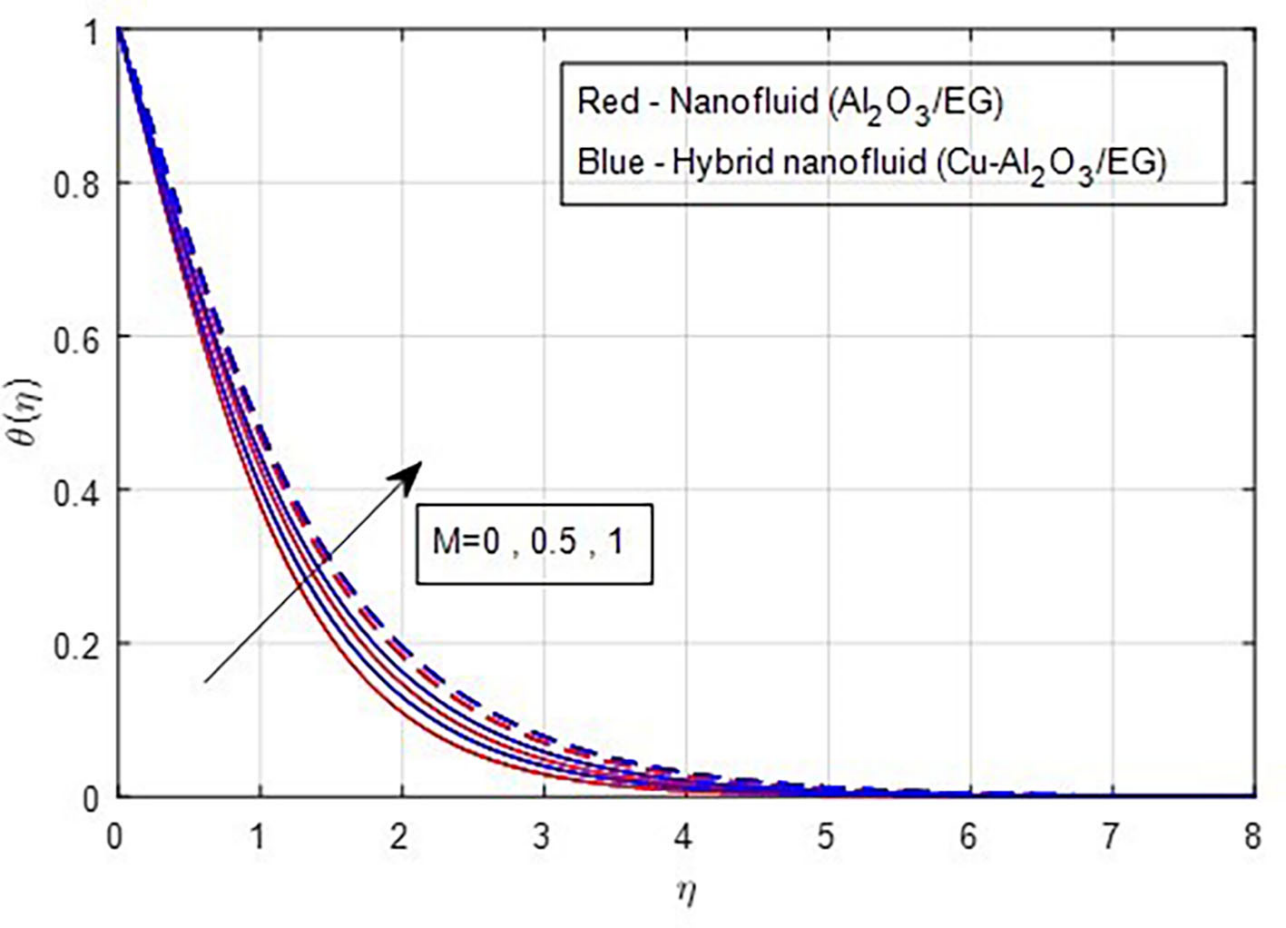
Temperature vs *M.*

**Figure 18.**
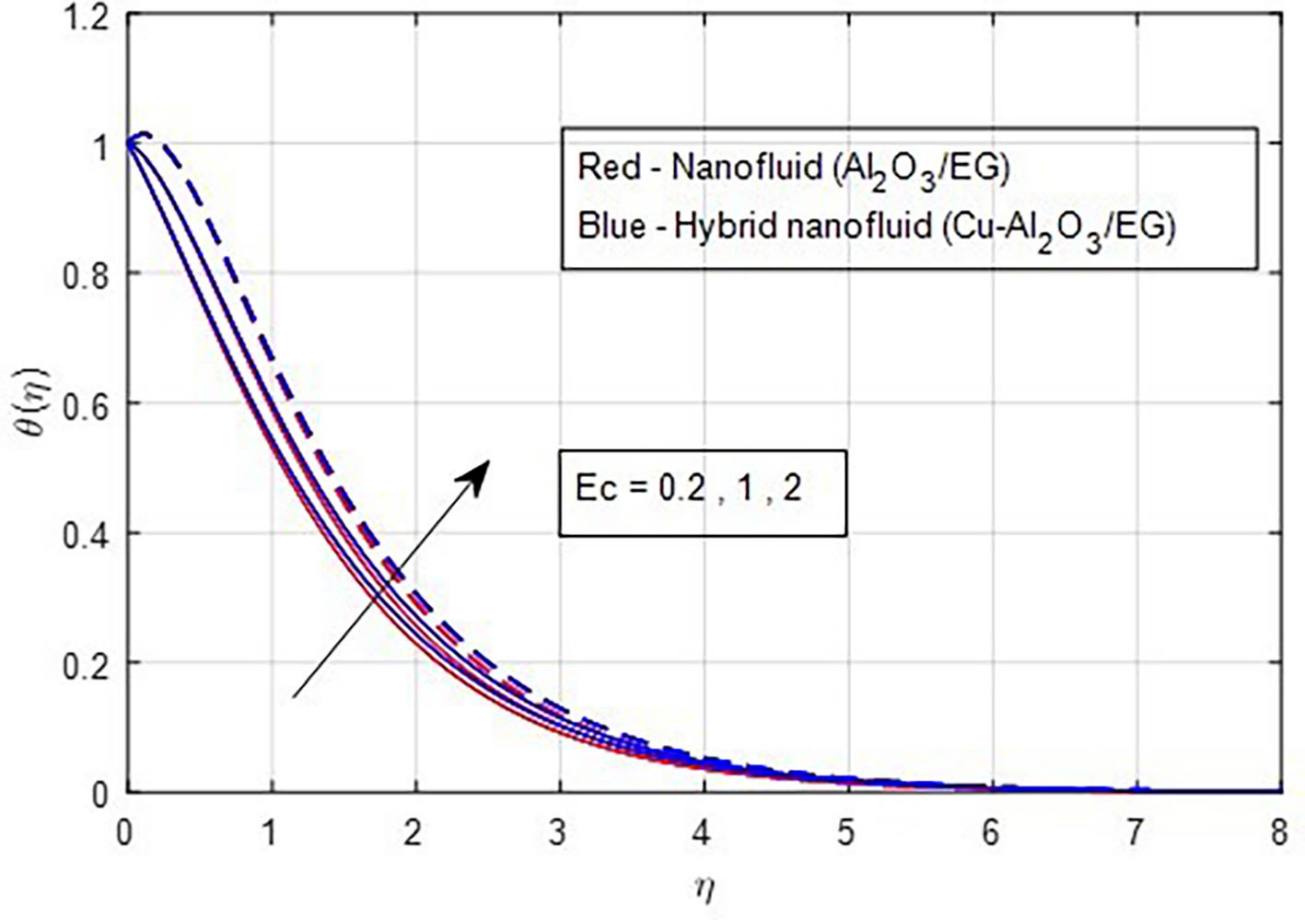
Temperature vs *Ec.*

**Figure 19.**
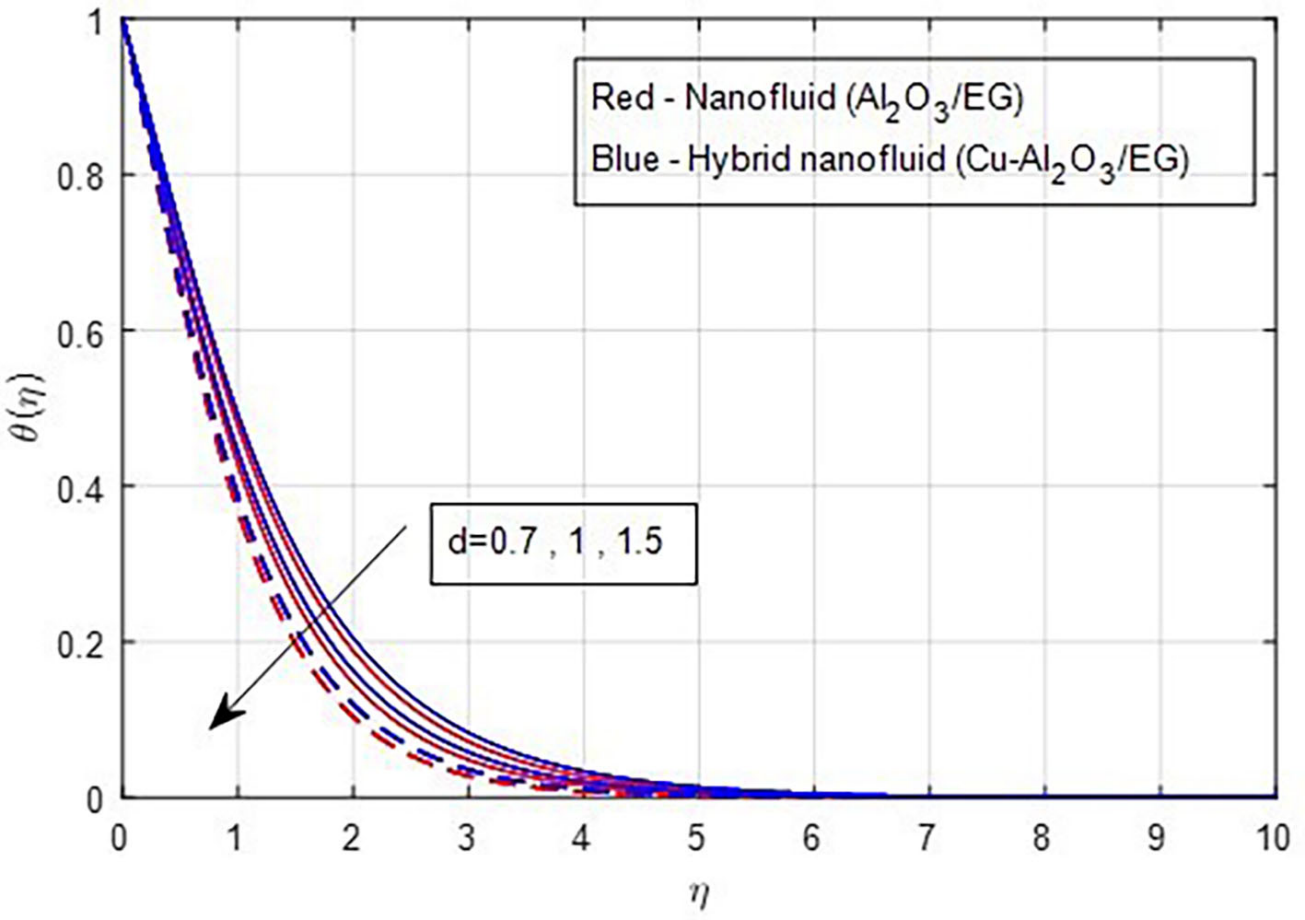
Temperature vs *d.*

**Figure 20.**
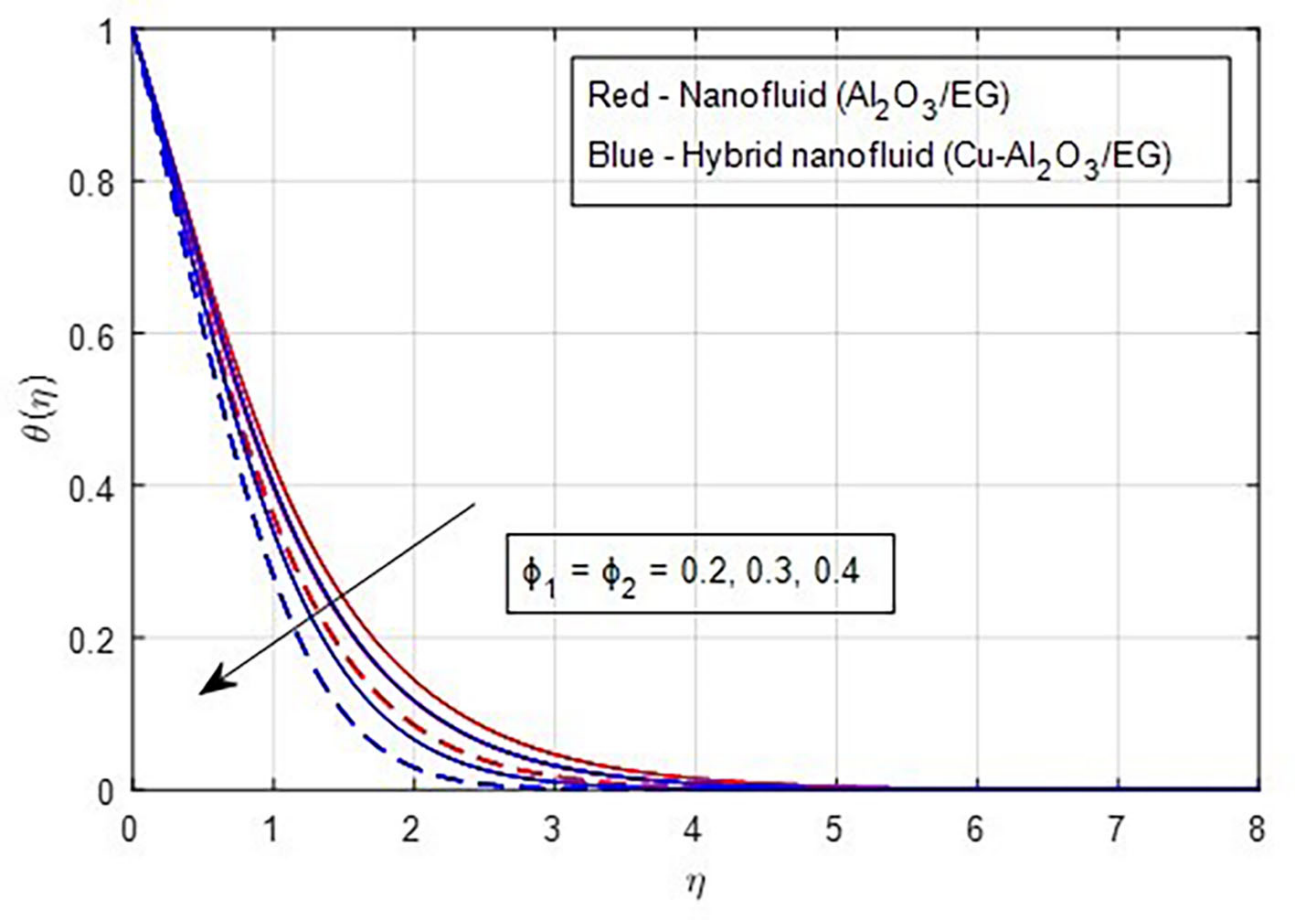
Temperature vs $\phi_{1},\phi_{2}.$

The impacts of the thermal relaxation parameter on fluid temperatures are illustrated in Figure 12. A greater thermal relaxation parameter occurs with temperature diminish. This trend can be explained by the fact that the thermal relaxation parameter in a non-Fourier heat transfer process quantifies the lag time between the temperature gradient and the flux. The time it takes for a material to return to a particular proportion of its equilibrium temperature following a temperature change is known as its thermal relaxation time. The temperature profile graph’s decline over time suggests that the material is gradually cooling. This is caused by the thermal relaxation time; the temperature will drop more slowly the higher the value. Figure 13 depicts the thermal distribution against R. The temperature of the thermal system rises when R increases because it increases thermal efficiency (conductivity). The temperature distribution and the accompanying thickness of the boundary film both improve with an increase in the R estimate. As a result, the temperature in the boundary layer region rises. In this instance, hybrid nanoparticles outperform mono-nanofluid. The strength of thermal boundaries increases as the source of heat generation grows. This physical process leads to increased heat transfer, which elevates the thermal profiles, as shown in Figure 14. The increase in thermal profiles is more pronounced for hybrid nanoparticles. The random motion of solid nanoparticles is directly related to higher values of the Brownian motion factor. As a result, the thermal boundary layer strengthens when the internal energy of the solid nanoparticles is converted into kinetic or heat energy. Consequently, the thermal properties rise as Brownian motion increases, as depicted in Figure 15. Similarly, when the thermophoresis factor increases, additional heat is transferred from a region of higher concentration to a region of lower concentration. Consequently, an increase in the thermophoresis parameter is correlated with a corresponding rise in the thermal properties, as Figure 16 illustrates. The temperature functions improve when the magnetic parameter M grows in value, as seen by the temperature distribution graphs in Figure 17. The valuable heat produced by the resistivity impact of the Lorentz force accounts for the rising thermal patterns observed for increasing values of magnetic parameters. These figures show that the curves produced by hybrid nanofluids are larger than those produced by mono-nanofluids. Hence, hybrid nanoparticles are noticed as more efficient at enhancing the base fluid temperature. The relationship between profiles of temperature and the Eckert number is seen in Figure 18, where it is observed that temperature profiles rise as the Eckert number grows. From a physical perspective, a higher Eckert number corresponds to a higher amount of heat energy in the boundary layer. Frictional heating keeps heat energy from being produced. As Figure 19 demonstrates, the temperature falls as the stretching ratio grows. We can observe that temperature reduces as the stretching ratio parameter increases. This could happen as a result of the fluid’s temperature dropping as a result of an increase in the stretching ratio parameter, which increases the flow of the colder fluid at the ambient surface toward the hot surface. Increases in the volumetric fraction of solid nanoparticles provide greater resistance to the flow of fluid because the fluid particles have higher densities. In this process, more heat is transmitted from the hotter zone to the colder zone. A graph of the temperature profile resulting from an increasing volume percentage of nanoparticles is presented in Figure 20. Physically, increasing the concentration of nanoparticles in the base fluid makes the fluid more viscous, leading to the formation of intermolecular frictional forces within the fluid. This results in a noticeable rise in the temperature curve at higher nanoparticle volume fractions. As a result, the temperature is observed to increase with a higher nanoparticle volume fraction.

### 4.3 Concentration characteristic

**Figure 21.**
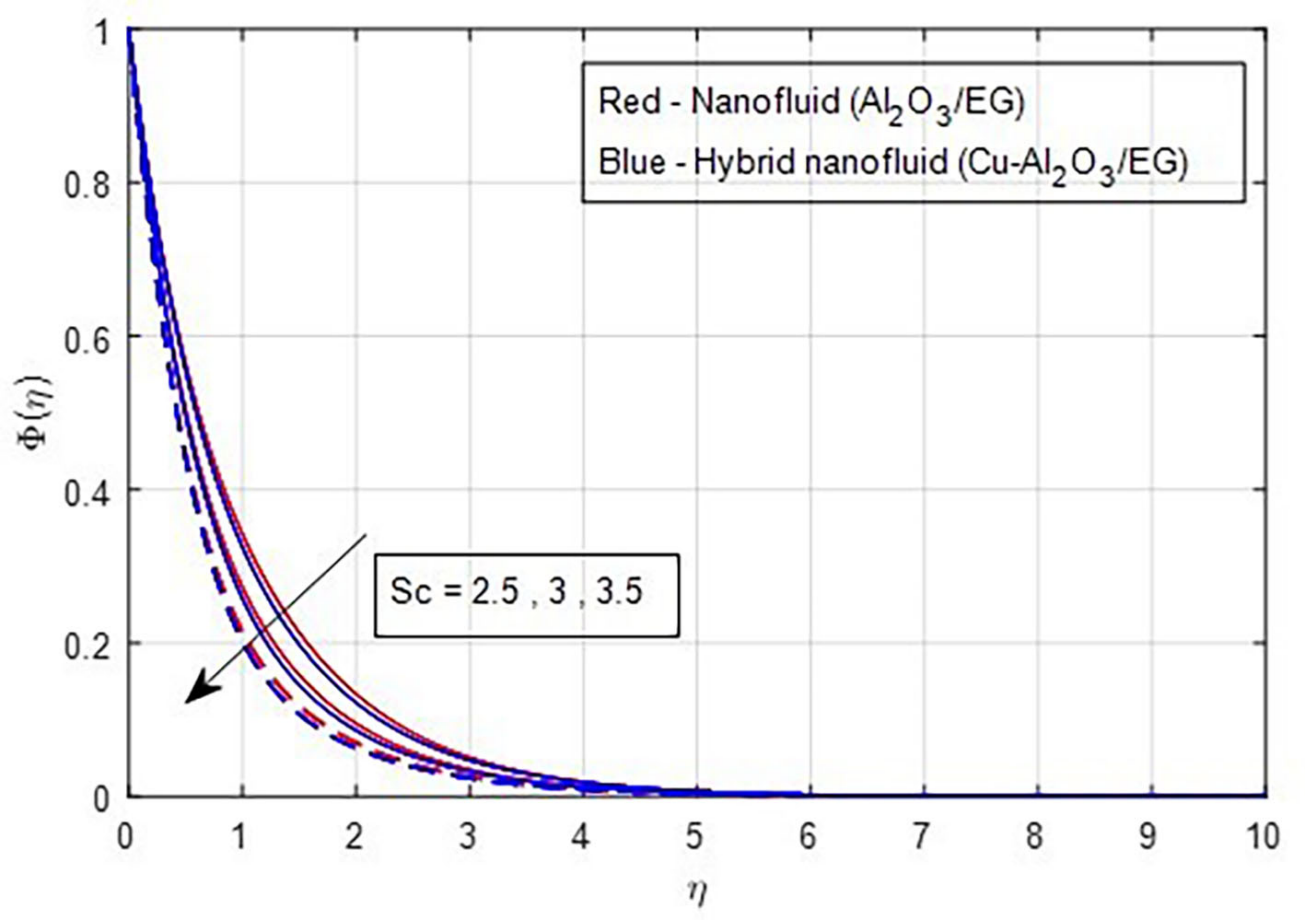
Concentration vs *Sc.*

**Figure 22.**
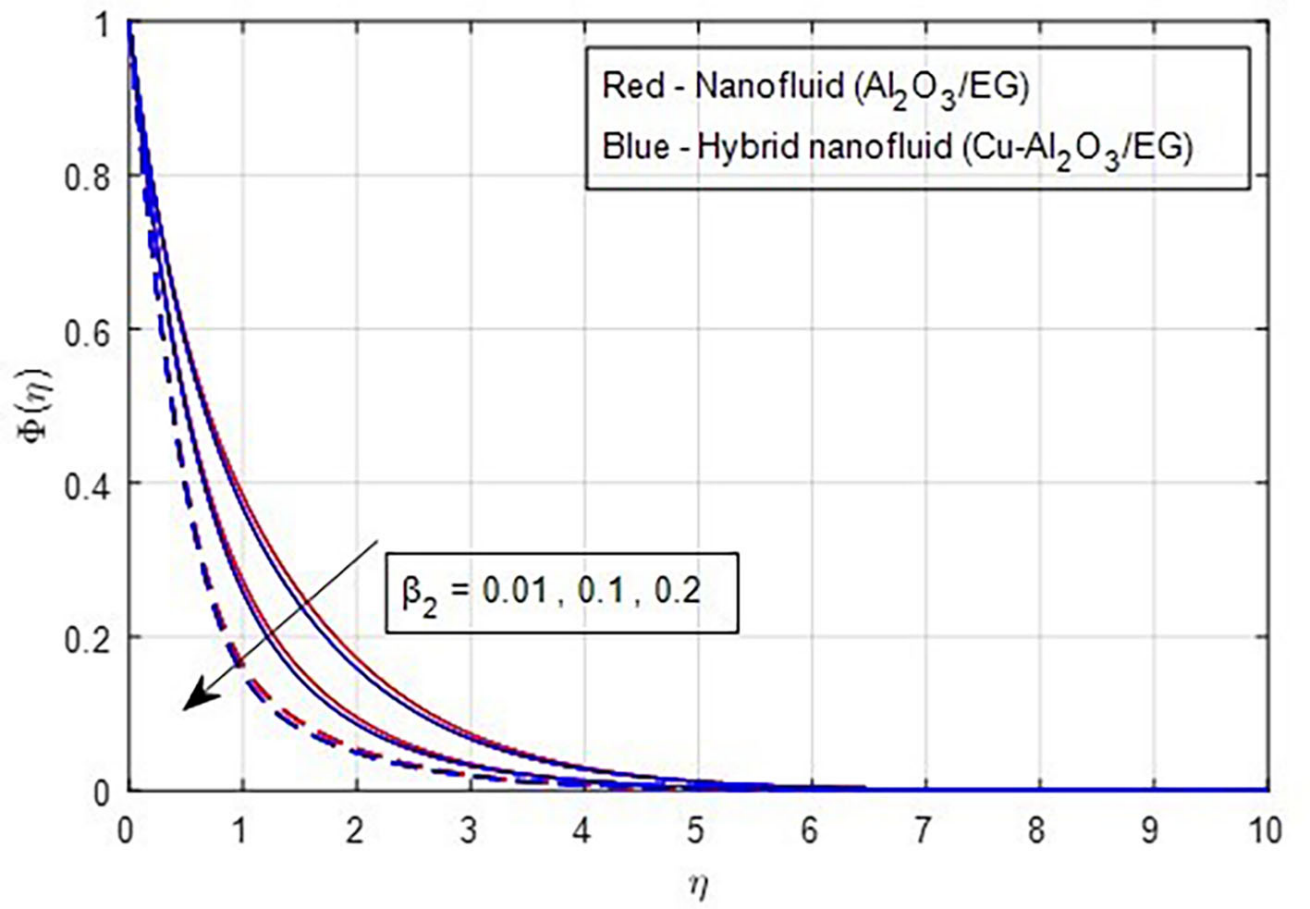
Concentration vs $\beta_{2}.$

**Figure 23.**
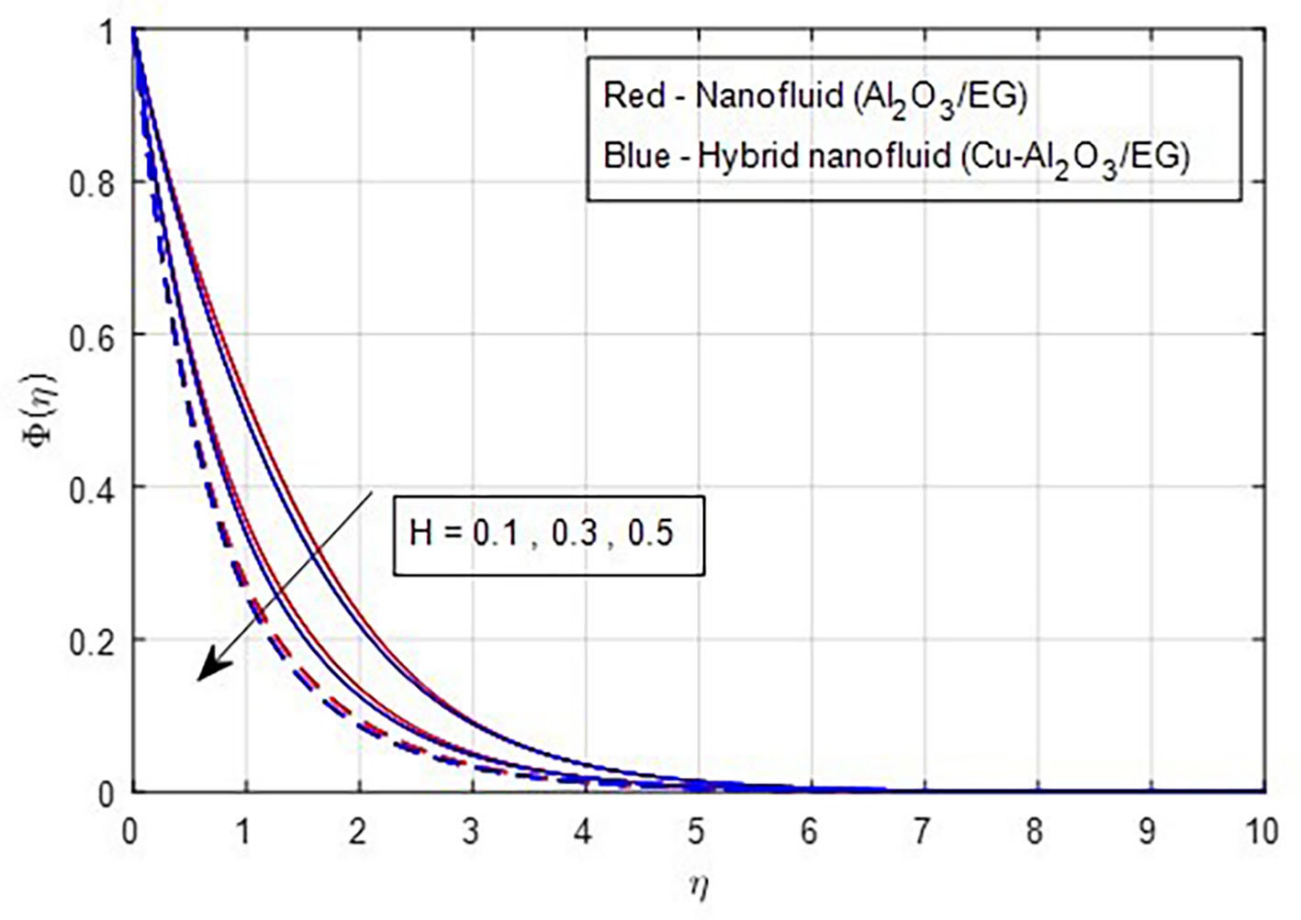
Concentration vs *H.*

**Figure 24.**
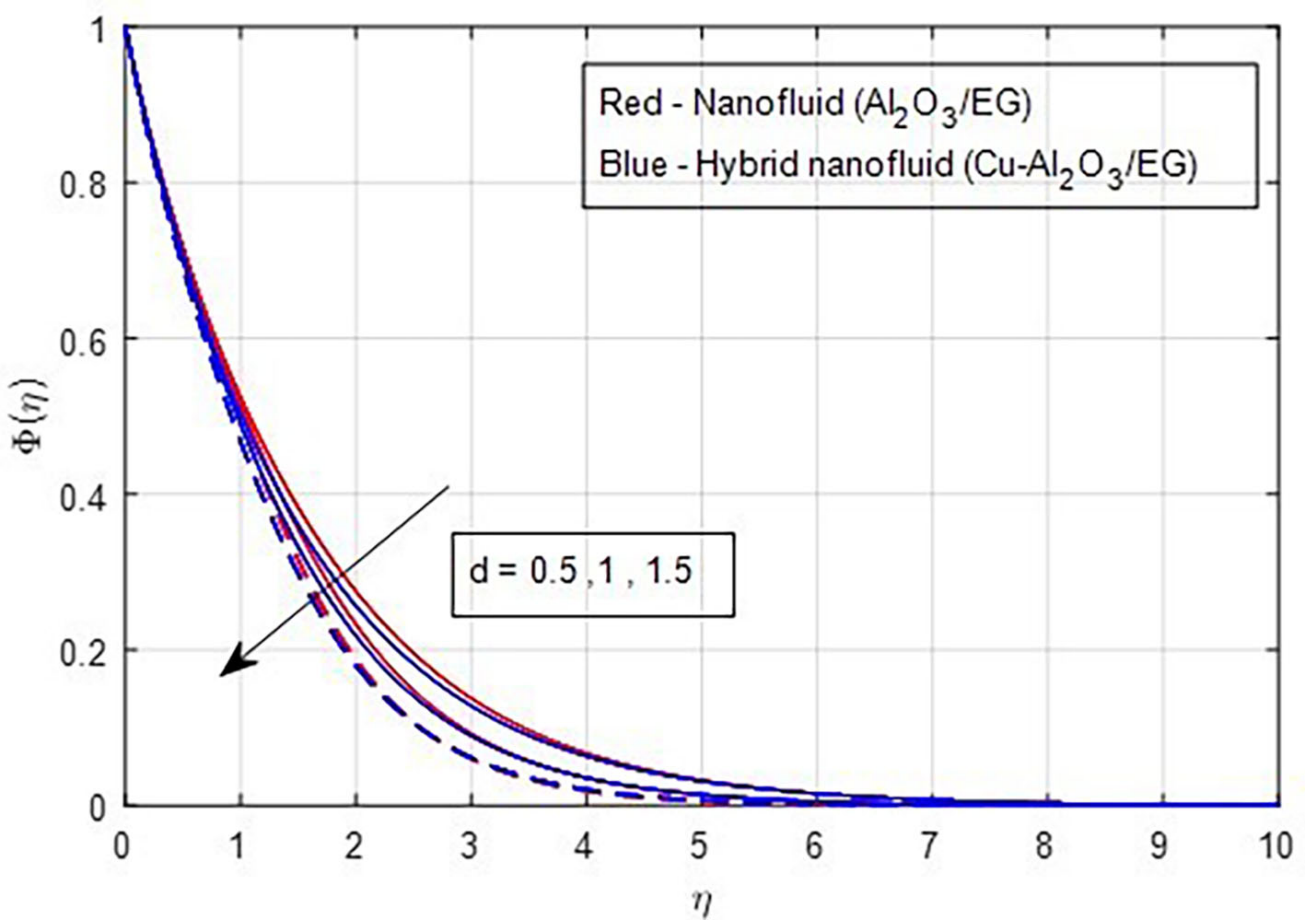
Concentration vs *d.*

Higher Schmidt numbers are linked to lower nanoparticle concentrations due to the inverse relationship between molecular diffusivity and the Schmidt number, as shown in Figure 21. Figure 22 depicts the effect of the concentration relaxation parameter on the concentration field. As the concentration relaxation parameter increases, both the concentration profile and the thickness of the concentration boundary layer decrease. This occurs because the particles need more time to diffuse when the concentration relaxation time parameter increases. Figure 23 shows that both the solute’s boundary layer thickness and the nanoparticle concentration in the hybrid nanofluid decrease as the chemical reaction parameter increases. Changes in the intensity of the chemical reaction affect the fluid’s diffusivity, leading to a reduction in concentration. Finally, Figure 24 illustrates the decrease in the concentration profile as the stretching ratio increases.

### 4.4 Influence of factors on Coefficient of skin friction, Nusselt and Sherwood numbers

In Table 3, the numerical results of skin friction drag along x- and y-directions (−CfxRe12) and (−d1.5CfyRe12) and the Nusselt number (NuxRe−12) , the Sherwood number (ShxRe−12) , the essential emerging parameters, are estimated using different dimensionless parameters for mono-nanofluids and hybrid nanofluids. For mono-nanofluids and hybrid nanofluids, skin friction drags rise in the x- and y-directions as $\phi_{1},\phi_{2},M$ , and d grow but diminish as We grows for mono-nanofluids and hybrid nanofluids. For hybrid nanofluids, Nusselt numbers grow with the values of $\phi_{1},\phi_{2},d$ and $\;\beta_{1}$ , but they decrease as $\text{We},M,\theta_{w},H,\beta_{2}$ , and R values increases. According to these findings, thermal radiation, temperature ratio parameters, magnetic forces, relaxation time, and other flow parameters all have a major impact on the rate of heat transfer close to the surface. Sherwood numbers drop with rising values of $\;d,\phi_{1},\phi_{2}$ , and $\beta_{1}$ , but increase with values of $\text{We},M,R,\theta_{w}$ , and H . These results indicate that the mass transfer rate near the surface is strongly affected by relaxation time, chemical processes, and other flow characteristics.

**Table 3.** Mathematical data of coefficient of skin friction, Nusselt and Sherwood number of some values of parameters.

*We*	*M*	$\phi_{1}$	$\phi_{2}$	*d*	β ^1^	*R*	θ *_w_*	$\beta_{2}$	*H*	−CfxRe12	−d32CfyRe12	NuxRe−12	ShxRe−12
0.1										1.939382	0.903738	0.488015	0.379954
0.2										1.873674	0.890098	0.478565	0.384044
0.3										1.794227	0.875349	0.467349	0.388856
	0									1.479947	0.653997	0.662693	0.284334
	1									1.873674	0.890098	0.478565	0.384044
	2									2.185542	1.073919	0.347022	0.454532
		0	0.01							1.461074	0.698628	0.421414	0.381833
		0	0.03							1.545986	0.740268	0.451952	0.375229
		0	0.05							1.634068	0.783370	0.484698	0.368544
		0.01	0.01							1.506024	0.718540	0.418845	0.385502
		0.03	0.03							1.684861	0.801975	0.446383	0.385221
		0.05	0.05							1.873674	0.890098	0.478565	0.384044
				0.1						1.814054	0.159463	0.342775	0.444855
				0.5						1.873674	0.890098	0.478565	0.384044
				1						1.940978	0.940978	0.588150	0.334435
					0.1					1.873674	0.890098	0.478565	0.384044
					0.2					1.873674	0.890098	0.489231	0.381149
					0.3					1.873674	0.890098	0.501131	0.377928
						0.2				1.873674	0.890098	0.478565	0.384044
						0.3				1.873674	0.890098	0.470320	0.403437
						0.4				1.873674	0.890098	0.464709	0.418902
							1.2			1.873674	0.890098	0.478565	0.384044
							1.4			1.873674	0.890098	0.416921	0.417224
							1.6			1.873674	0.890098	0.347853	0.452785
								0.1		1.873674	0.890098	0.478565	0.384044
								0.2		1.873674	0.890098	0.477401	0.409733
								0.3		1.873674	0.890098	0.476151	0.439006
									0.2	1.873674	0.890098	0.478565	0.384044
									1	1.873674	0.890098	0.467503	1.012973
									2	1.873674	0.890098	0.464065	1.449359

Mathematical data of coefficient of skin friction, Nusselt and Sherwood number of some values of parameters for *d* = 0 *.*5, *Nb* = *Nt* = $\theta_{w}$ = *Q* = *K* = β 1 = β 2 = 0 *.*1 *,We* = *H* = *R* = *Ec* = 0 *.*2, *Pr* = *M* = *Sc* = 1 *,* $\phi_{1}$ = $\phi_{2}$ = 0 *.*05.

## 5. Conclusion

This section presents the results for a 3D MHD Williamson hybrid nanofluid flow. The study utilizes the Cattaneo-Christov and modified Boungerno’s models to simulate the Cu−Al _2_O _3_/Ethylene glycol MHD Williamson hybrid nanofluid flow over a linearly stretching sheet, influenced by factors such as magnetic field, heat generation/absorption, non-linear thermal radiation, Joule heating, Brownian motion, thermophoresis, porosity, Williamson fluid parameter, Eckert number, Prandtl number, Schmidt number, chemical reaction parameter, stretching ratio, thermal relaxation, and concentration relaxation parameters. By applying appropriate similarity variables, the system of partial differential equations is transformed into a non-linear ordinary differential equation formulation. These ordinary differential equations are solved numerically using the bvp4c solver on the MATLAB platform. Similarly, graphical analysis is used to plot the obtained flow parameters so that their effects on flow, heat, and mass can be easily seen. Researchers and engineers working on related issues can benefit greatly from the obtained data. Moreover, our findings demonstrate the effectiveness and utility of hybrid nanofluid performance.

The present study leads to the following deductions:

• When the stretching ratio parameter rises, the primary velocity profile diminishes, but the secondary velocity profile grows.
• The primary and secondary velocities diminishes with increasing porosity, the Williamson fluid, and the magnetic field parameter.
• As the volume fractions of mono-nanofluid and hybrid nanofluid increase, both the primary and secondary velocities decrease. The addition of nanoparticles to a base fluid enhances the viscous forces, resulting in a reduction of the fluid’s velocity. However, the primary and secondary velocity profiles of the Williamson hybrid nanofluid model are notably higher compared to those of the mono-nanofluid and conventional base fluid models.
• When the non-linear thermal radiation parameter, heat generation ( *Q*_0_ *>* 0), Brownian motion, thermophoresis, magnetic field parameter, and Eckert number increases, the temperature profile rises. The temperature profile diminishes, when the stretching ratio and thermal relaxation parameter increases.
• Temperature curves diminishes with increases estimates of nanofluid volume fraction and hybrid nanofluid volume fraction. When nanoparticles are added to a base fluid, the viscosity increases and the fluid temperature falls. When viscosity increases, temperature decreases.
• As the stretching ratio, concentration relaxation, chemical reaction parameter, and Schmidt number rise, the concentration profile decreases.
• As the volume fraction of hybrid nanoparticles increases, both the skin friction and Nusselt number of the Williamson hybrid nanofluid rise. However, the local Sherwood number of the MHD Williamson hybrid nanofluid decreases with a higher volume fraction of hybrid nanoparticles.
• Finally, based on the current findings, we deduced that hybrid nanofluid performs better in ethylene glycol than mono-nanofluid.

A dedicated section on future research directions follows the conclusion, outlining potential extensions of this study. These include examining unsteady flow conditions to capture transient effects, exploring various hybrid nanoparticle combinations to enhance heat transfer performance, and extending the model to incorporate non-Newtonian rheological properties relevant to biomedical and industrial applications.

## CRediT authorship contribution statement

Asfaw Tsegaye Moltot contributed to Writing review & editing, conceptualization, methodology, formal analysis, validation, and writing the original draft. Eshetu Haile contributed to Writing the review, editing, supervision, conceptualization, and resources. Gurju Awgichew contributed to Writing the review, editing, supervision and resources. Hunegnaw Dessie contributed to Writing the review, editing, supervision and resources.

## Ethics and consent

Ethical approval and consent were not required.

## Data Availability

BVP-MATLAB-Implementation and Thermophysical properties: https://data.mendeley.com/datasets/s4447nmmmr/2. ^58^ This project contains the following data:
- Values of physical parameter and Matlab Code Tan_22: MATLAB Implementation [Data set]. Zenodo. https://doi.org/10.5281/zenodo.14480542. ^59^ Data are available under the terms of the Creative Commons Attribution 4.0 International license (CC-BY 4.0).

## Software availability

• Source code available from: A list of detailed material properties used for algorithms and thermophysical properties of nanoparticle model analysis was taken from: https://github.com/asfawmat/BVP-MATLAB-Implementation ^60^ ( https://github.com/asfawmat/BVP-MATLAB-Implementation ^60^) for Matlab implementation.
• Archived software available from: https://doi.org/10.5281/zenodo.14480542 ^59^
• License: Data are available under the terms of the Creative Commons Attribution 4.0 International license (CC-BY 4.0).
