# Supplementary material for: Effect of nonlinear thermal radiation and Cattaneo-Christov heat and mass fluxes of Williamson hybrid nanofluid over a stretching porous sheet
Source: F1000Res. 2025 Dec 3;14:210. Originally published 2025 Feb 14. [Version 3] doi: 10.12688/f1000research.160734.3 (PMC12090890; doi:10.12688/f1000research.160734.3)
Supplement: Peer review history

# Reviewer report: Reviewer response for version 3

**DOI:** 10.5256/f1000research.192060.r438380

**Published:** 2025-12-24

**Authors:** Dr. Muhammad Bilal

**Affiliations:** 1 The University of Chenab, Gujrat, Pakistan

All the comments are addressed, and the article can be published now.

Is the work clearly and accurately presented and does it cite the current literature?

Yes

If applicable, is the statistical analysis and its interpretation appropriate?

Not applicable

Are all the source data underlying the results available to ensure full reproducibility?

Yes

Is the study design appropriate and is the work technically sound?

Yes

Are the conclusions drawn adequately supported by the results?

Yes

Are sufficient details of methods and analysis provided to allow replication by others?

Yes

Reviewer Expertise:

Computational Fluid Dynamics

I confirm that I have read this submission and believe that I have an appropriate level of expertise to confirm that it is of an acceptable scientific standard.

# Reviewer report: Reviewer response for version 2

**DOI:** 10.5256/f1000research.179489.r374557

**Published:** 2025-04-29

**Authors:** Ephesus Olusoji Fatunmbi

**Affiliations:** 1 Federal Polytechnic, Ilaro, Nigeria

The authors have revised the manuscript as suggested in my review comments. Thus, the  article can be accepted for indexing.

Is the work clearly and accurately presented and does it cite the current literature?

Yes

If applicable, is the statistical analysis and its interpretation appropriate?

Not applicable

Are all the source data underlying the results available to ensure full reproducibility?

Partly

Is the study design appropriate and is the work technically sound?

Partly

Are the conclusions drawn adequately supported by the results?

Yes

Are sufficient details of methods and analysis provided to allow replication by others?

Yes

Reviewer Expertise:

Applied mathematics, Computational fluid dynamics, Nano fluid, Boundary layer flow, etc.

I confirm that I have read this submission and believe that I have an appropriate level of expertise to confirm that it is of an acceptable scientific standard.

# Reviewer report: Reviewer response for version 2

**DOI:** 10.5256/f1000research.179489.r374718

**Published:** 2025-04-28

**Authors:** Dr. Muhammad Faisal

**Affiliations:** 1 The University of Azad Jammu and Kashmir, Muzaffarabad, Pakistan

In this manuscript, copper (Cu) and aluminum oxide (Al₂O₃) nanoparticles are suspended in ethylene glycol (C₂C₆O₂) to form the hybrid nanofluid. The governing partial differential equations are transformed into ordinary differential equations using similarity transformations and solved numerically with MATLAB’s bvp4c solver. The study examines various parameters, including stretching ratio, nanoparticle volume fraction, and relaxation times for concentration and thermal effects. Results are validated against existing literature.

 1. There are many typos and grammatical errors in the manuscript. Improve the English level.

 2. The title of the manuscript is too long. Revise the title.

 3. Provide references to those equations that are extracted from literature.

 4. What are the properties of the Williamson fluid, and in what category of non-Newtonian fluid does it lie? Explain in the manuscript.

 5. Provide the vector forms of governing equations, and also include a reference for boundary conditions.

 6. The mathematical expressions for the involved engineering parameters are missing.

 7. What are the limitations of the applied numerical technique?

 8. The value of the Prandtl number is missing in the investigation. Include its value and its plot?

 9. Provide a Nomenclature section.

 10. What are the reference velocity and reference temperature in this fluid flow model?

 11. Discuss novel findings in the abstract, providing relevant applications.

 12. Provide the rate of convergence of the solution obtained from the numerical method.

 13. Improve the introduction section by incorporating the latest studies. Some suggested works are given below:

 On Maxwell slip flow of radiative ternary hybrid nanofluid subject to Smoluchowski-Nield’s constraints using an iterative numerical simulation. Simulation of Casson hybrid nanofluid over bidirectional stretching surface with entropy analysis in stagnated domain. Semi-analytical simulation of chemically reactive Maxwell nanofluid with Cattaneo-Christov heat and mass fluxes. Insight into the bidirectional dynamics of hyperbolic tangent hybrid nanomaterial subject to Nield’s conditions using a numerical approach. Rivlin fluid flow between rotatable stretching disks with Cattaneo Christov heat transport.  Stagnation point flow of third-order nanofluid towards a lubricated surface using hybrid homotopy analysis method. Nanoparticles hybridization in bidirectional flowing of Prandtl-Eyring material with temperature-dependent conductivity: A numerical approach. Prescribed thermal effects on the dynamics of radiative and chemically reactive hyperbolic tangent nanofluid. Entropic behavior with activation energy in the dynamics of hyperbolic-tangent mixed convective nanomaterial due to a vertical slendering surface. Natural convective behavior of hybrid nanofluid (Al2O3-Cu/water) in an isosceles triangular cavity with bifurcation analysis. Entropic behavior in bidirectional flow of CeO2-ZnO/water hybrid nanofluid with prescribed surface temperature/heat flux aspects. Darcy-Forchheimer dynamics of hybrid nanofluid due to porous Riga surface capitalizing Cattaneo-Christov theory. Entropy optimization in squeezed nanofluidic dissipative transport of radiative water conveying aluminum alloys. Dynamics of MHD tangent hyperbolic nanofluid with prescribed thermal conditions, random motion, and thermo-migration of nanoparticles.

 11. A robust validation is needed.

 Given the above observations, a minor revision is requested before a final decision.

Is the work clearly and accurately presented and does it cite the current literature?

Partly

If applicable, is the statistical analysis and its interpretation appropriate?

Yes

Are all the source data underlying the results available to ensure full reproducibility?

Yes

Is the study design appropriate and is the work technically sound?

Yes

Are the conclusions drawn adequately supported by the results?

Yes

Are sufficient details of methods and analysis provided to allow replication by others?

Yes

Reviewer Expertise:

Fluid Mechanics, Computational Fluid Dynamics, Heat and Mass Transfer

I confirm that I have read this submission and believe that I have an appropriate level of expertise to confirm that it is of an acceptable scientific standard, however I have significant reservations, as outlined above.

# Response

**Published:** 2025-11-22

**Authors:** Asfaw Tsegaye

**Affiliations:** Mathematics, Bahir Dar University, Bahir Dar, Amhara, Ethiopia

**Author’s Response to Reviewer’s 4 Comments**

 We sincerely appreciate the reviewers for their insightful and constructive comments, which have significantly improved the quality of our manuscript. Below, we provide our responses to each of the reviewer’s concerns and outline the corresponding revisions made in the manuscript.

1. There are many typos and grammatical errors in the manuscript. Improve the English level.

**Author’s reply: **Thank you for this observation. The entire manuscript has been thoroughly revised for grammar, clarity, and academic writing quality. All typographical and linguistic errors have been corrected throughout the text.

       2. The title of the manuscript is too long. Revise the title.

**Author’s reply: **We appreciate the suggestion. The title has been shortened and refined to improve clarity and conciseness.

       3. Provide references to those equations that are extracted from literature.

**Author’s reply: **Thank you for pointing this out. Appropriate references have now been added to all equations derived or adapted from existing literature.

       4. What are the properties of the Williamson fluid, and in what category of non-Newtonian fluid does it lie? Explain in the manuscript.

**Author’s reply: **We have added a detailed explanation in the introduction. The Williamson fluid model is a **shear-thinning (pseudoplastic) non-Newtonian fluid**, and its rheological characteristics and constitutive equations are now clearly described with supporting citations.

       5. Provide the vector forms of governing equations, and also include a reference for boundary conditions.

**Author’s reply: **We have included the full vector forms of the governing momentum, heat, and mass equations, along with references supporting the selected boundary conditions.

        6. The mathematical expressions for the involved engineering parameters are missing.

**Author’s reply: **These expressions have now been added, particularly for the skin-friction coefficients, the Nusselt number, and the Sherwood number.

        7. What are the limitations of the applied numerical technique?

**Author’s reply: **We have added a paragraph in the numerical method section discussing limitations of the **bvp4c** approach, including sensitivity to initial guesses, computational cost, and potential difficulty in handling highly stiff systems.

        8. The value of the Prandtl number is missing in the investigation. Include its value and its plot?

**Author’s reply: **The Prandtl number has been included in the parameter tables, and an additional figure has been added to show its influence on the temperature field.

        9. Provide a Nomenclature section.

**Author’s reply: **A complete Nomenclature section has been added immediately after the abstract.

       10. What are the reference velocity and reference temperature in this fluid flow model?

**Author’s reply: **The reference velocity and reference temperature definitions have been added and clearly explained in the problem formulation section.

        11. Discuss novel findings in the abstract, providing relevant applications.

**Author’s reply: **The abstract has been revised to highlight the novelty of the study, especially the combined effects of nonlinear radiation, Cattaneo-Christov fluxes, and 3D Williamson hybrid nanofluid flow, and to outline practical applications in cooling systems, polymer manufacturing, and energy technologies.

        12. Provide the rate of convergence of the solution obtained from the numerical method.

**Author’s reply: **A convergence table showing the rate of convergence of the **bvp4c** solver has been added to the numerical method section.

        13. Improve the introduction section by incorporating the latest studies. Some suggested works are given below: On Maxwell slip flow of radiative ternary hybrid nanofluid subject to Smoluchowski-Nield’s constraints using an iterative numerical simulation. Simulation of Casson hybrid nanofluid over bidirectional stretching surface with entropy analysis in stagnated domain. Semi- analytical simulation of chemically reactive Maxwell nanofluid with Cattaneo-Christov heat and mass fluxes. Insight into the bidirectional dynamics of hyperbolic tangent hybrid nanomaterial subject to Nield’s conditions using a numerical approach. Rivlin fluid flow between rotatable stretching disks with Cattaneo-Christov heat transport. Stagnation point flow of third-order nanofluid towards a lubricated surface using hybrid Homotopy analysis method. Nanoparticles hybridization in bidirectional flowing of Prandtl-Eyring material with temperature-dependent conductivity: A numerical approach. Prescribed thermal effects on the dynamics of radiative and chemically reactive hyperbolic tangent nanofluid. Entropic behavior with activation energy in the dynamics of hyperbolic-tangent mixed convective nanomaterial due to a vertical slandering surface. Natural convective behavior of hybrid nanofluid (Al2O3-Cu/water) in an isosceles triangular cavity with bifurcation analysis. Entropic behavior in bidirectional flow of CeO2- ZnO/water hybrid nanofluid with prescribed surface temperature/heat flux aspects. Darcy- Forchheimer dynamics of hybrid nanofluid due to porous Riga surface capitalizing Cattaneo- Christov theory. Entropy optimization in squeezed nanofluidic dissipative transport of radiative water conveying aluminum alloys. Dynamics of MHD tangent hyperbolic nanofluid with prescribed thermal conditions, random motion, and thermo-migration of nanoparticles.

**Author’s reply: **Following the reviewer’s recommendations, the introduction has been significantly expanded to include all the suggested recent works on Maxwell slip flow, Casson hybrid nanofluid, third-order nanofluid, tangent hyperbolic fluids, Prandtl-Eyring materials, and entropy analysis–based studies.

         14. A robust validation is needed.

**Author’s reply: **A new validation subsection has been added. Table 2 compares our results with existing literature, demonstrating excellent agreement and confirming the accuracy of our numerical scheme.

 All revisions have been incorporated into the manuscript, enhancing clarity, technical rigor, and alignment with reviewer feedback. We appreciate the opportunity to improve our work.

 Sincerely,

 Asfaw Tsegaye Moltot [Corresponding Author]

 Email: asfawmat@gmail.com

# Reviewer report: Reviewer response for version 2

**DOI:** 10.5256/f1000research.179489.r374558

**Published:** 2025-04-23

**Authors:** Nandagopal K

**Affiliations:** 1 Mohan Babu University, Tirupathi, India

The author satisfies all the comments. So I recommend that the manuscript is indexed.

Is the work clearly and accurately presented and does it cite the current literature?

Yes

If applicable, is the statistical analysis and its interpretation appropriate?

Yes

Are all the source data underlying the results available to ensure full reproducibility?

Yes

Is the study design appropriate and is the work technically sound?

Yes

Are the conclusions drawn adequately supported by the results?

Partly

Are sufficient details of methods and analysis provided to allow replication by others?

Yes

Reviewer Expertise:

Fluid dynamics , peristalsis, porous medium

I confirm that I have read this submission and believe that I have an appropriate level of expertise to confirm that it is of an acceptable scientific standard.

# Reviewer report: Reviewer response for version 2

**DOI:** 10.5256/f1000research.179489.r374716

**Published:** 2025-04-23

**Authors:** Dr. Muhammad Bilal

**Affiliations:** 1 The University of Chenab, Gujrat, Pakistan

This article focuses on the flow of a Williamson hybrid nanofluid over a stretching porous sheet, incorporating the effects of non-linear thermal radiation and Cattaneo–Christov heat and mass fluxes. The article is suitable for indexed after addressing the following minor revisions:

1. There are some grammatical errors in the manuscript, particularly in the **Introduction** section. The authors are advised to thoroughly proofread the manuscript and correct these errors.
2. Please provide the **units** of all physical quantities listed in the **Nomenclature** section.
3. Although the **Introduction** is generally well written, the authors are encouraged to strengthen it further by incorporating some **recent studies**.
4. **Figure 1** should be redrawn. Kindly remove the background and clearly indicate the key physical aspects of the flow geometry for better visualization.
5. Ensure that all displayed **equations** are punctuated appropriately—insert **commas or full stops** at the end as needed.
6. In **Equation (23)**, please correct the **Eckert number symbols** along the x- and y-directions to reflect accurate notation.
7. The definition of the **Weissenberg number** includes the variable xxx, which typically challenges the validity of a similarity solution. The authors should briefly justify how similarity is preserved in this context.
8. In the **first-order system of equations**, all terms involving “A” must include appropriate **subscripts** for clarity and precision.

Is the work clearly and accurately presented and does it cite the current literature?

Yes

If applicable, is the statistical analysis and its interpretation appropriate?

Not applicable

Are all the source data underlying the results available to ensure full reproducibility?

Yes

Is the study design appropriate and is the work technically sound?

Yes

Are the conclusions drawn adequately supported by the results?

Yes

Are sufficient details of methods and analysis provided to allow replication by others?

Yes

Reviewer Expertise:

Computational Fluid Dynamics

I confirm that I have read this submission and believe that I have an appropriate level of expertise to confirm that it is of an acceptable scientific standard, however I have significant reservations, as outlined above.

# Response

**Published:** 2025-11-22

**Authors:** Asfaw Tsegaye

**Affiliations:** Mathematics, Bahir Dar University, Bahir Dar, Amhara, Ethiopia

**Author’s Response to Reviewer’s 3 Comments**

 We sincerely appreciate the reviewers for their insightful and constructive comments, which have significantly improved the quality of our manuscript. Below, we provide our responses to each of the reviewer’s concerns and outline the corresponding revisions made in the manuscript.

1. There are some grammatical errors in the manuscript, particularly in the introduction section. The authors are advised to thoroughly proofread the manuscript and correct these errors.

**Author’s reply: **The manuscript requires a comprehensive revision to address numerous grammatical and typographical errors present throughout the text. Careful proofreading is necessary to enhance the overall clarity and readability of the paper.

 2. Please provide the units of all physical quantities listed in the Nomenclature section.

**Author’s reply: **The nomenclature section has been updated to include SI units for all

            parameters.

 3. Although the Introduction is generally well-written, the authors are encouraged to strengthen it further by incorporating some recent studies.

**Author’s reply: **The references have been incorporated into the introduction and literature review to contextualize and support our research.

**For instance,**

                  -  Novel features of radiating hybrid nanofluid flow past a nonlinear stretchable porous sheet with different nanoparticle shapes. 

                  -  Computation of Convective Magnetohydrodynamic Buongiorno Nanofluid Transport from an Inclined Plane with Ion Slip And Hall Current Effects.

                  -  Influence of Darcy–Forchheimer Fe 3 O 4-CoFe 2 O 4-H 2 O hybrid nanofluid flow with magnetohydrodynamic and viscous dissipation effects past a permeable stretching sheet: numerical contribution.

 4. Figure 1 should be redrawn. Kindly remove the background and clearly indicate the key physical aspects of the flow geometry for better visualization.

**Author’s reply: **Thank you for the suggestion. Figure 1 has been redrawn to improve clarity. The background has been removed, and key physical aspects of the flow geometry such as the stretching sheet, coordinate axes, and flow direction are now clearly labeled to enhance visualization and understanding.

 5. Ensure that all displayed equations are punctuated appropriately—insert commas or full stops at the end as needed.

**Author’s reply: **Corrected

 6. In Equation (23), please correct the Eckert number symbols along the x- and y-directions to reflect accurate notation.

**Author’s reply: **Corrected

 7. The definition of the **Weissenberg number** includes the variable xxx, which typically challenges the validity of a similarity solution. The authors should briefly justify how similarity is preserved in this context.

**Author’s reply: **Justification:****

          We acknowledge that the inclusion of the spatial variable x in the definition of the Weissenberg number (We) can, in general, pose a challenge to the existence of the similarity solution. However, in our study, the functional dependence of We on x is such that it varies in a manner consistent with the chosen similarity transformation. Specifically, the definition of We is constructed to preserve the invariance of the transformed equations when expressed in terms of the similarity variable η. This allows the partial differential equations to be reduced to ordinary differential equations, ensuring the validity of the similarity solution. Similar formulations can be found in prior literature dealing with viscoelastic or non-Newtonian flows in boundary layer settings, where such transformations have been successfully applied under analogous assumptions.

 8. In the **first-order system of equations**, all terms involving “A” must include appropriate **subscripts** for clarity and precision.

**Author’s reply: **It is modified in the revised manuscript.

 All revisions have been incorporated into the manuscript, enhancing clarity, technical rigor, and alignment with reviewer feedback. We appreciate the opportunity to improve our work.

 Sincerely,

 Asfaw Tsegaye Moltot [Corresponding Author]

 Email: asfawmat@gmail.com

# Reviewer report: Reviewer response for version 1

**DOI:** 10.5256/f1000research.176670.r366915

**Published:** 2025-03-04

**Authors:** Nandagopal K

**Affiliations:** 1 Mohan Babu University, Tirupathi, India

This study explores the Effect of non-linear thermal radiation and Cattaneo-Christov heat and mass fluxes on the flow of Williamson hybrid nanofluid over a stretching porous sheet.

 However it requires some following corrections. The work can be suggested to indexed after the following corrections are fulfilled.

 1. The whole article may be checked for typo and  other grammatical mistakes.

 2. Justification must be provided on the use of a particular set of boundary conditions. Why   

     these  specific sets of conditions are suitable for  this study?

 3. What are the advantages of BVP4C over other  methods?

 4.Justify the selection of transformation to reduce the PDEs into ODEs.

 5. Discussion should be improved, and it should be  based on the physical reasoning of the model. Some quantitative analysis must support the  results.    

 6.  Add Nomenclature separately

 7. ****What is the significance of studying non-linear thermal radiation?

 8.   Add some result for stream lines to make their  study sound more unique.

 9.  What is  the difference between thermal radiation and non-linear thermal radiation?

 10. How current results present directions to the  future studies. Summarize in conclusion section.

 11. Authors must re-check and update the   introduction section. Include some recent  literature survey subject to the current title.  Moreover, remove old-fashioned studies  with latest research. Refer 1 to 6. 

 The present research work may be suggested to publish in F1000 research after the following corrections are fulfilled.

Is the work clearly and accurately presented and does it cite the current literature?

Yes

If applicable, is the statistical analysis and its interpretation appropriate?

Yes

Are all the source data underlying the results available to ensure full reproducibility?

Yes

Is the study design appropriate and is the work technically sound?

Yes

Are the conclusions drawn adequately supported by the results?

Partly

Are sufficient details of methods and analysis provided to allow replication by others?

Yes

Reviewer Expertise:

Fluid dynamics , peristalsis, porous medium

I confirm that I have read this submission and believe that I have an appropriate level of expertise to confirm that it is of an acceptable scientific standard, however I have significant reservations, as outlined above.

## References

[1] : Numerical treatment of entropy generation in convective MHD Williamson nanofluid flow with Cattaneo–Christov heat flux and suction/injection. *International Journal of Modelling and Simulation* .2024; 10.1080/02286203.2024.2405714 1-18 10.1080/02286203.2024.2405714

[2] : Analysis of Entropy Optimization in MHD Flow of Non-Newtonian Nanofluids with Chemical Reaction and Thermal Energies. *Journal of Computational and Theoretical Transport* .2024; 10.1080/23324309.2024.2419008 1-26 10.1080/23324309.2024.2419008

[3] : Activation of energy in MHD Casson nanofluid flow through a porous medium in the presence of convective boundary conditions and suction/injection. *Numerical Heat Transfer, Part A: Applications* .2023; 10.1080/10407782.2023.2271655 1-17 10.1080/10407782.2023.2271655

[4] : Convective flow of MHD non-Newtonian nanofluids on a chemically reacting porous sheet with Cattaneo-Christov double diffusion. *Waves in Random and Complex Media* .2022; 10.1080/17455030.2022.2111478 1-20 10.1080/17455030.2022.2111478

[5] : Higher Order Chemical Reaction and Radiation Effects on Magnetohydrodynamic Flow of a Maxwell Nanofluid With Cattaneo–Christov Heat Flux Model Over a Stretching Sheet in a Porous Medium. *Journal of Fluids Engineering* .2022;144(4) : 10.1115/1.4053250 10.1115/1.4053250

[6] : MHD effect on peristaltic motion of Williamson fluid via porous channel with suction and injection. *Partial Differential Equations in Applied Mathematics* .2025;13: 10.1016/j.padiff.2025.101103 10.1016/j.padiff.2025.101103

# Response

**Published:** 2025-03-15

**Authors:** Asfaw Tsegaye

**Affiliations:** Mathematics, Bahir Dar University, Bahir Dar, Amhara, Ethiopia

**Author’s Response to Reviewers’ Comments**

 We sincerely appreciate the reviewers for their insightful and constructive comments, which have significantly improved the quality of our manuscript. Below, we provide our responses to each of the reviewer’s concerns and outline the corresponding revisions made in the manuscript.

 1. The whole article may be checked for typos and other grammatical mistakes.

**Author’s reply: **We have carefully reviewed the manuscript and corrected all typographical and grammatical errors to ensure clarity and readability.

 2. Justification must be provided on the use of a particular set of boundary conditions. Why these specific sets of conditions are suitable for this study?

**Author’s reply: ** The specific boundary conditions used in this study are justified based on the physical and mathematical framework of the problem:

 a. Physical Relevance:

 - The boundary conditions at *z=0(w=0, v=* *v* *w* *=by, u=* *u* *w* *=ax,  T=* *T* *w* *, C=* *C* *w* *)*​ represent a stretching

    sheet, which is common in industrial applications such as polymer extrusion, metal forming, and         

    coating processes.

 -  The prescribed velocity components *(* *u* *w* *=ax,* *v* *w* *=by)* mimic the stretching motion of the sheet in two 

    directions, ensuring a controlled boundary layer development.

 b. Mathematical Consistency:

 -  At the sheet ( *z=0)*, specifying velocity ensures that the no-slip condition is satisfied, which is 

    fundamental for boundary layer flow.

 -  The temperature and concentration at the sheet *(* *T* *w* *,* *C* *w* *)*​ are held constant to analyze the effects of

    heat and mass transfer within the hybrid nanofluid.

 c. Far-Field Conditions:

 -  As *z→∞*, the velocity components *(u, v)* approach zero, ensuring that the fluid is at rest far from 

    the sheet.

 -  The temperature and concentration also reach ambient conditions *(* *T* *∞* *,* *C* *∞* *)*, signifying thermal and 

     concentration equilibrium.

 d. Applicability to Cattaneo-Christov Model:

 -  The use of Cattaneo-Christov heat and mass flux models requires that thermal and concentration

     relaxation effects be properly accounted for at the boundaries.

 -  The conditions ensure a well-posed problem, allowing numerical solutions via similarity

     transformations and MATLAB’s **bvp4c** solver.

 Overall, these boundary conditions are essential for accurately capturing the physics of the problem while ensuring numerical stability and consistency with previous studies.

 3. What are the advantages of BVP4C over other methods?

**Author’s reply: **The **BVP4C** method in MATLAB offers several advantages over other numerical methods for solving boundary value problems:

 a. High Accuracy:

 -  The **BVP4C** solver utilizes a finite difference method with collocation techniques, achieving an 

     accuracy of approximately *10* *-7*. This makes it highly precise for solving boundary layer flow 

     problems.

 b. Built-in Error Control:

**-   BVP4C** automatically adjusts the mesh selection and step size based on residual error estimates,

      ensuring numerical stability and reducing computational errors.

 c. Efficient Handling of Singularities:

 -   It can handle singular boundary conditions effectively, which is particularly useful in fluid dynamics 

      problems involving **stretching sheets**, hybrid nanofluids, and thermal radiation effects.

 d. Adaptive Mesh Refinement:

 -   Unlike shooting methods, which require iterative guessing, **BVP4C** refines the solution using an 

     adaptive mesh, leading to more efficient computations without divergence issues.

 e. Well-Suited for Stiff Equations:

 -   Since boundary layer problems often involve **stiff** differential equations, **BVP4C** efficiently handles

      them without requiring excessive computational power.

 f. Direct Implementation of Boundary Conditions:

 -   Unlike shooting methods, where unknown initial conditions need estimation, **BVP4C** directly

     incorporates boundary conditions, making it more robust for complex problems​

 4. Justify the selection of transformation to reduce the PDEs into ODEs.

**Author’s reply: **The selection of similarity transformations to reduce the partial differential equations (PDEs) into ordinary differential equations (ODEs) is justified based on several key factors:

 a. Reduction of Complexity

 -   The governing equations of the hybrid nanofluid flow problem are highly nonlinear PDEs. Using

      similarity transformations simplifies these equations into a set of ODEs, making them more 

      manageable for numerical or analytical solutions.

 b. Boundary Layer Theory

 -   The similarity variable *η=z* *a* *ν* *f*  is chosen to convert the spatial dependency of the velocity,

      temperature, and concentration into a single variable. This transformation is in line with classical

      boundary layer theory, which seeks to reduce multi-dimensional flow problems into a one-

      dimensional form for efficient analysis.

 c. Consistency with Existing Literature

 -   The chosen transformations align with prior studies on fluid flow over stretching sheets, ensuring that 

      the results can be compared with previous findings and validated accordingly.

 d.  Mathematical and Computational Efficiency

 -    The resulting system of ODEs is more suitable for numerical techniques like MATLAB’s **bvp4c**, which

       is specifically designed for solving boundary value problems with high accuracy and stability.

 e. Physical Relevance

 -   The transformed equations retain the fundamental physical characteristics of the original PDEs, 

     capturing key effects like thermal radiation, hybrid nanofluid properties, and the Cattaneo-Christov

     heat and mass flux model.By using these similarity transformations, the study ensures that the

     problem formulation is both mathematically rigorous and computationally efficient.

 5. Discussion should be improved, and it should be based on the physical reasoning of the model. Some

     quantitative analysis must support the results.

**Author’s reply: **The discussion section has been revised to provide deeper insights into the physical mechanisms governing hybrid nanofluid flow. We have elaborated on the effects of dimensionless parameters on velocity, temperature, and concentration fields, supported by quantitative analysis and comparison with existing studies.

 6. Add Nomenclature separately

**Author’s reply: **A separate nomenclature section has been included to define symbols and parameters used throughout the study for better readability.

 7. What is the significance of studying non-linear thermal radiation?

**Author’s reply: **Studying non-linear thermal radiation is essential for accurately modeling heat transfer in high-temperature environments, as it accounts for complex interactions beyond the Stefan-Boltzmann Law. It plays a crucial role in applications like aerospace, nuclear reactors, and industrial heat exchangers, where extreme temperatures amplify radiative effects. Non-linear models enhance heat flux predictions, particularly in nanofluid-based systems, where radiation significantly influences thermal behavior. In hybrid nanofluids like Cu-Al₂O₃/ethylene glycol, it affects boundary layer characteristics and heat dissipation efficiency. Understanding these effects helps optimize thermal management systems, improving cooling performance in electronics, solar panels, and combustion chambers. Additional discussion on its significance has been included in the introduction section.

 8. Add some result for streamlines to make their study sound more unique.

**Author’s reply: **We have outlined future research directions on results for plotting streamlines and will continue exploring this topic in upcoming studies.

 9. What is the difference between thermal radiation and non-linear thermal radiation?

**Author’s reply: **Thermal radiation follows the **Stefan-Boltzmann law**, where heat flux is proportional to the fourth power of temperature and is often linearized under small temperature variations. In contrast, **non-linear thermal radiation** arises in high-temperature environments where heat flux exhibits a more complex dependence on temperature, influenced by **temperature gradients, material optical properties, and medium characteristics**. This non-linearity makes it more accurate for modeling extreme heat transfer conditions, such as those found in **nanofluids, aerospace applications, and combustion chambers**. ****We have added a clarification in the introduction section.

 10. How current results present directions to the future studies. Summarize in conclusion section.

**Author’s reply: **The current study highlights key factors influencing hybrid nanofluid flow, providing directions for future research. Further studies can explore optimized stretching conditions, alternative nanoparticle materials, and advanced thermal models, including non-linear radiation effects. Investigating unsteady flow, non-Newtonian fluids, and three-dimensional effects will enhance the findings' applicability in engineering and biomedical fields.

 11. Authors must re-check and update the introduction section. Include some recent literature survey subject to the current title. Moreover, remove old-fashioned studies with latest research. Refer 1 to 6.

 - Reddy M, Vajravelu K, Ajithkumar M, Sucharitha G, et al.: Numerical treatment of entropy generation in convective MHD Williamson nanofluid flow with Cattaneo–Christov heat flux and suction/injection. International Journal of Modelling and Simulation. 2024. 1-18 Publisher Full Text

 -M. S. I, Lakshminarayana P, Sucharitha G, Vinodkumar Reddy M, et al.: Analysis of Entropy Optimization in MHD Flow of Non-Newtonian Nanofluids with Chemical Reaction and Thermal Energies. Journal of Computational and Theoretical Transport. 2024. 1-26 Publisher Full Text

 - Vinodkumar Reddy M, Lakshminarayana P, Vajravelu K, Sucharitha G: Activation of energy in MHD Casson nanofluid flow through a porous medium in the presence of convective boundary conditions and suction/injection. Numerical Heat Transfer, Part A: Applications. 2023. 1-17 Publisher Full Text -

 -  Vinodkumar Reddy M, Sucharitha G, Vajravelu K, Lakshminarayana P: Convective flow of MHD non-Newtonian nanofluids on a chemically reacting porous sheet with Cattaneo-Christov double diffusion. Waves in Random and Complex Media. 2022. 1-20 Publisher Full Text

 -  Vinodkumar Reddy M, Lakshminarayana P: Higher Order Chemical Reaction and Radiation Effects on Magnetohydrodynamic Flow of a Maxwell Nanofluid With Cattaneo–Christov Heat Flux Model Over a Stretching Sheet in a Porous Medium. Journal of Fluids Engineering. 2022; 144 (4). Publisher Full Text - 

 -   Chakradhar K, Nandagopal K, Prashanthi V, Parandhama A, et al.: MHD effect on peristaltic motion of Williamson fluid via porous channel with suction and injection. Partial Differential Equations in Applied Mathematics. 2025; 13. Publisher Full Text

**Author’s reply: **We have incorporated the suggested references in the introduction and literature review, explaining their relevance to our work. These studies provide context for our analysis and strengthen the foundation of the manuscript.

 All revisions have been incorporated into the manuscript, enhancing clarity, technical rigor, and alignment with reviewer feedback. We appreciate the opportunity to improve our work.

 Sincerely,

 Asfaw Tsegaye Moltot[Corresponding Author]

 Email: asfawmat@gmail.com

# Reviewer report: Reviewer response for version 1

**DOI:** 10.5256/f1000research.176670.r366913

**Published:** 2025-02-28

**Authors:** Ephesus Olusoji Fatunmbi

**Affiliations:** 1 Federal Polytechnic, Ilaro, Nigeria

Reviewer comments on the manuscript titled “ **Effect of non-linear thermal radiation and Cattaneo-Christov heat and mass fluxes on the flow of Williamson hybrid nanofluid over a stretching porous sheet”**

**The following are my comments on this manuscript:**

 1). The manuscript introduction lacks basic ingredients to convince the readers about the importance of the study. In the last paragraph of the introduction, the authors should clearly identify and state areas of application for the study. More so, the existing gap which the authors intend to fill should be clearly stated.

 2). The assumptions for modeling the problems are not adequately stated in the manuscript. Authors should justify the inclusion of various terms in the governing equations and give details of their derivation with appropriate references.

 3). Why the choice of the nanoparticles and base fluid used in the study? It is important to state the significance and applications of the nanoparticles used.

 4). Why the choice of the method of solution? Include the importance and limitations of this method in the study.

 5). The results and discussion can be improved by including basic scientific reasons for the trend exhibited by various parameters on the flow fields.

 6). Include the future scope of study after the conclusion section.

Is the work clearly and accurately presented and does it cite the current literature?

Yes

If applicable, is the statistical analysis and its interpretation appropriate?

Not applicable

Are all the source data underlying the results available to ensure full reproducibility?

Partly

Is the study design appropriate and is the work technically sound?

Partly

Are the conclusions drawn adequately supported by the results?

Yes

Are sufficient details of methods and analysis provided to allow replication by others?

Yes

Reviewer Expertise:

Applied mathematics, Computational fluid dynamics, Nano fluid, Boundary layer flow, etc.

I confirm that I have read this submission and believe that I have an appropriate level of expertise to confirm that it is of an acceptable scientific standard, however I have significant reservations, as outlined above.

# Response

**Published:** 2025-03-15

**Authors:** Asfaw Tsegaye

**Affiliations:** Mathematics, Bahir Dar University, Bahir Dar, Amhara, Ethiopia

**Author’s Response to Reviewers’ Comments**

 We sincerely appreciate the reviewers for their insightful and constructive comments, which have significantly improved the quality of our manuscript. Below, we provide our responses to each of the reviewer’s concerns and outline the corresponding revisions made in the manuscript.

 1. The manuscript introduction lacks basic ingredients to convince the readers about the importance of the study. In the last paragraph of the introduction, the authors should clearly identify and state areas of application for the study. More so, the existing gap which the authors intend to fill should be clearly stated.

**Author’s reply: **The introduction has been revised to clearly highlight the significance of hybrid nanofluids in industrial applications such as cooling systems, biomedical engineering, and energy systems.  The last paragraph of the introduction explicitly identifies the research gap by emphasizing that no prior studies have investigated the combined effects of **non-linear thermal radiation, Cattaneo-Christov heat and mass fluxes, Joule heating, and chemical reactions** on Williamson hybrid nanofluid flow over a stretching porous sheet. The findings of this study have significant implications in various **engineering and industrial applications**, including aerospace engineering, nuclear power plants, electronic cooling systems, biomedical applications, and polymer and textile industries.

 2. The assumptions for modeling the problems are not adequately stated in the manuscript. Authors should justify the inclusion of various terms in the governing equations and give details of their derivation with appropriate references.

**Author’s reply: **Thank you for your suggestions. A dedicated section has been added to justify the assumptions made in modeling the problem. The inclusion of specific terms in the governing equations (such as Steady-State, Three-Dimensional Flow, Williamson Non-Newtonian Fluid Model, Cattaneo-Christov Heat and Mass Flux Model, Non-Linear Thermal Radiation, Joule Heating, Viscous Dissipation, Porous Medium and Magnetic Field Effects, heat generation, chemical reaction terms, and Boundary Conditions) is now explicitly explained with appropriate references. Additional citations have been included to validate the selection of mathematical models for hybrid nanofluids.

 3. Why the choice of the nanoparticles and base fluid used in the study? It is important to state the significance and applications of the nanoparticles used.

**Author’s reply: **This study utilizes a Cu-Al₂O₃/Ethylene Glycol hybrid nanofluid, chosen for its superior thermal properties, stability, and industrial relevance. Copper (Cu) enhances thermal and electrical conductivity, making it ideal for electronics and automotive cooling, while Aluminum Oxide (Al₂O₃) offers stability, corrosion resistance, and broad industrial applications. Ethylene Glycol serves as the base fluid due to its widespread use in cooling systems, wide temperature stability, and effective nanoparticle dispersion. This hybrid nanofluid is applicable in aerospace, nuclear reactors, biomedical engineering, electronic cooling, and renewable energy systems, making it an optimal choice for advanced heat transfer applications.

 4. Why the choice of the method of solution? Include the importance and limitations of this method in the study.

**Author’s reply: **In this study, the MATLAB BVP4C solver is used to solve the transformed

 ordinary differential equations (ODEs) derived from the governing partial differential equations (PDEs). BVP4C is chosen for its high accuracy, adaptive mesh refinement, and robustness in handling boundary value problems (BVPs), making it well-suited for nonlinear and coupled ODEs arising in Williamson hybrid nanofluid flow. Unlike shooting methods, BVP4C ensures stability and efficiently manages multi-point boundary conditions, making it ideal for fluid flow and heat transfer problems. While alternative methods like Runge-Kutta and finite difference approaches were considered, they were found to be less efficient due to stability and convergence issues. However, BVP4C’s computational intensity requires careful selection of initial guesses.

 5. The results and discussion can be improved by including basic scientific reasons for the trend exhibited by various parameters on the flow fields.

**Author’s reply: **The revised discussion section now offers a detailed scientific interpretation of velocity, temperature, and concentration trends, supported by physical laws and real-world applications. Each parameter's impact is analyzed with **scientific reasoning and quantitative analysis**, and comparisons with existing literature are included to validate the findings.

 6. Include the future scope of the study after the conclusion section.

**Author’s reply: **A dedicated section on future research directions follows the conclusion, outlining potential extensions of this study. These include examining unsteady flow conditions to capture transient effects, exploring various hybrid nanoparticle combinations to enhance heat transfer performance, and extending the model to incorporate non-Newtonian rheological properties relevant to biomedical and industrial applications.

 All revisions have been incorporated into the manuscript, enhancing clarity, technical rigor, and alignment with reviewer feedback. We appreciate the opportunity to improve our work.

 Sincerely,

 Asfaw Tsegaye Moltot[Corresponding Author]
